# Multi-faceted analysis provides little evidence for recurrent whole-genome duplications during hexapod evolution

**DOI:** 10.1186/s12915-020-00789-1

**Published:** 2020-05-27

**Authors:** Dick Roelofs, Arthur Zwaenepoel, Tom Sistermans, Joey Nap, Andries A. Kampfraath, Yves Van de Peer, Jacintha Ellers, Ken Kraaijeveld

**Affiliations:** 1 Department of Ecological Science, Vrije Universiteit, De Boelelaan 1085, 1081HV Amsterdam, The Netherlands; 2 Keygene N.V, Agro Business Park 90, 6708 PW Wageningen, The Netherlands; 3 Center for Plant Systems Biology, VIB, B-9052 Ghent, Belgium; 4 Department of Plant Biotechnology and Bioinformatics, Ghent University, B-9052 Ghent, Belgium; 5 Department of Biochemistry, Genetics and Microbiology, Center for Microbial Ecology and Genomics, University of Pretoria, Pretoria, 0028 South Africa; 6 Origins Center, Nijenborgh 7, 9747AG Groningen, The Netherlands; 7 Institute for Biodiversity and Ecosystem Dynamics, University of Amsterdam, Sciencepark 904, 1090 GE Amsterdam, The Netherlands

**Keywords:** Polyploidy, Gene duplication and loss, Co-linearity, Insecta, Collembola, Gene tree reconciliation, Synonymous distance

## Abstract

**Background:**

Gene duplication events play an important role in the evolution and adaptation of organisms. Duplicated genes can arise through different mechanisms, including whole-genome duplications (WGDs). Recently, WGD was suggested to be an important driver of evolution, also in hexapod animals.

**Results:**

Here, we analyzed 20 high-quality hexapod genomes using whole-paranome distributions of estimated synonymous distances (*K*_*S*_), patterns of within-genome co-linearity, and phylogenomic gene tree-species tree reconciliation methods. We observe an abundance of gene duplicates in the majority of these hexapod genomes, yet we find little evidence for WGD. The majority of gene duplicates seem to have originated through small-scale gene duplication processes. We did detect segmental duplications in six genomes, but these lacked the within-genome co-linearity signature typically associated with WGD, and the age of these duplications did not coincide with particular peaks in *K*_*S*_ distributions. Furthermore, statistical gene tree-species tree reconciliation failed to support all but one of the previously hypothesized WGDs.

**Conclusions:**

Our analyses therefore provide very limited evidence for WGD having played a significant role in the evolution of hexapods and suggest that alternative mechanisms drive gene duplication events in this group of animals. For instance, we propose that, along with small-scale gene duplication events, episodes of increased transposable element activity could have been an important source for gene duplicates in hexapods.

## Background

Gene duplication is an important source of genetic variation that can propel adaptive evolution and speciation [1, 2]. Large-scale gene duplication events, such as large segmental or whole-genome duplications (WGDs), are thought to have played a major role in evolution because they supply hundreds or even thousands of novel gene duplicates on which evolution can work. Such events enhance evolutionary innovation due to the creation of genetic redundancy [3], may increase mutational and environmental robustness [4], and reduce the risk of extinction [5, 6]. WGD events have also been linked to increased diversification [7, 8], either directly or after a lag-time period [7, 9], but see [10]. It has also been argued that WGD may facilitate adaptation and survival under specific conditions, for instance during periods of environmental turmoil or cataclysmic events [11]. In animals, WGD has been rarely detected, for which various explanations have been put forward, such as different reproductive modes [12], dosage-sensitive sex determination [12–14], and more intricate physiological and developmental constraints [12]. Nevertheless, ancient WGDs are hypothesized to have played a role in the evolution of teleost fish, mollusks [15], and particularly arthropods [16–18].

**Fig. 1:**
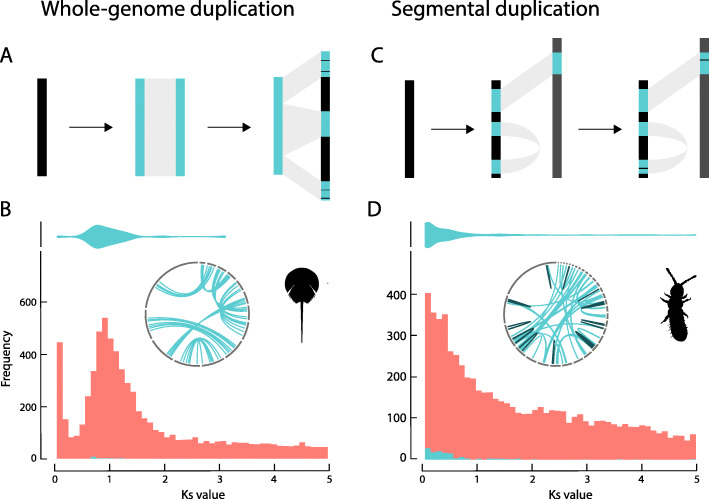
Co-linear patterns and *Ks* histograms illustrating differences between whole-genome duplication and putative bursts of segmental duplications observed in this study: **a** conceptual co-linearity pattern of (ancient) whole-genome duplication; black represents ancestral chromosome; light blue represents a duplicated chromosome containing gene duplicates with similar synonymous divergence (*Ks*). Genome rearrangements will fragment duplicated chromosomes into syntenic blocks. **b***Ks* frequency distribution of *L. polyphemus* (an arthropod that underwent WGD). Orange-red, histogram of the node-weighted whole-paranome *Ks* distribution; light-blue violin plot and histogram, *Ks* distribution of gene duplicates anchored in co-linear blocks as inferred from MCScanX; inlay, circos plot of co-linear blocks: all duplicate segments reside on separate scaffolds. **c** conceptual co-linearity pattern of a burst of segmental duplication; black represents an ancestral chromosome; light blue represents duplicated segments. Duplicated segments may end up on a position in another chromosome (gray bar), or on the same chromosome giving rise to tandem repeats and palindromes graphically represented as arches on a single scaffold. A burst of segmental duplication is hypothesized if the *Ks* values of gene pairs anchored in co-linear blocks are not clustered in a distinctive *Ks* peak corresponding to a peak in the whole-paranome distribution. **d***Ks* histogram and circos plot of co-linear blocks of gene duplicates from *F. candida*. Orange-red, *Ks* histogram using all gene duplicates; light-blue violin plot and histogram, *Ks* distribution for gene duplicates anchored in co-linear blocks. Inlay, circos plot showing co-linear block distribution among and within scaffolds. See “Results” for further interpretation. Note that the older a potential genome duplication event, the more difficult it is to discriminate between alternative explanations. Silhouettes are derived from phylopic.org

Arthropods are a highly speciose and diverse group of animals. Gene and genome duplications may have played an important role in generating this diversity, yet patterns of duplication in this group are still under discussion. With the recent availability of high-quality invertebrate genome sequences, several cases of large-scale gene duplication and potential WGDs have been identified. For example, genomes of arachnids were found to harbor many paralogous gene pairs and a duplicated Hox gene cluster, indicating a WGD event in their evolutionary history [19, 20]. The horseshoe crab *Limulus polyphemus* even shows four copies of the Hox gene cluster, suggesting two rounds of WGD within this group [16, 20]. Recently, Li et al. [18] reported 18 ancient WGDs and six other large-scale bursts of gene duplication in 118 analyzed transcriptomes and 25 genomes of hexapods. The inferred pattern of scattered WGDs across the phylogenetic tree of hexapods would indicate that WGDs have been an important driver of evolutionary novelty and diversity in insects. However, inference of ancient WGDs remains challenging [21]. For instance, in a recent study, some of us showed that unaccounted variation in duplication and loss rates across lineages can strongly affect assessment of the presence or absence of WGDs [22]. Also, a recent reanalysis of the *Bombyx mori* data could not confirm a previously reported putative Lepidoptera-specific WGD [23]. Therefore, it remains unclear whether the observed patterns of duplications in hexapods are indeed indicative of frequent ancient WGDs. To reliably distinguish the presence of remnants of ancient WGD from alternative scenarios, several independent lines of evidence are required. One line of evidence is peaks in *K*_S_ distributions (Fig. 1a, b), where the number of duplication events is plotted as a function of an estimate of the synonymous distance associated with these events (*K*_S_, which serves as a proxy for age). Bursts of duplicates with similar synonymous divergence are indicative of a large-scale duplication event, although one has to be aware of the caveats in interpreting such distributions [21, 24, 25]. For one, *K*_S_ distributions cannot be used to infer very ancient WGDs, due to saturation of the synonymous distance and the stochastic nature of the molecular clock [24].

An important second line of evidence for uncovering remnants of large-scale or whole-genome duplications is within-genome co-linearity (Fig. 1a, b). In the absence of gene loss and rearrangements associated with the rediploidization process, we expect duplicated pairs (referred to as “anchor pairs” or “anchors”) to initially reside in syntenic and co-linear blocks (Fig. 1a). Genome rearrangements and gene loss will erode this signal over time, but even for ancient duplication events, substantial intragenomic co-linearity remains observable [26–29]. Such intragenomic co-linear blocks are usually assumed to result from ancient WGD, although other events, such as bursts of transposon activity, translocations, or aneuploidy, can also potentially generate similar signals (Fig. 1b) [30]. Furthermore, very ancient WGDs can often no longer be reliably identified from co-linear analyses, in particular when high-quality chromosome-level assemblies are lacking. In those cases, lastly, most evidence is based on the analysis of gene trees (e.g., [18, 31, 32]). However, assessing the support for a hypothetical WGD from gene trees remains very challenging, and results from such approaches should be treated with considerable caution [22, 33]. Generally, the combination of temporal (from *K*_S_ distributions and gene trees) and structural (co-linearity) evidence provides the most reliable means towards distinguishing WGD from other sources of gene duplication, yet requires high-quality genome data across multiple species.

Here, we present a multi-faceted analysis of highly contiguous, well-annotated genomes of 20 hexapod species and one outgroup, *Limulus polyphemus*, to study the occurrence of gene- and genome duplication events in this diverse species group. To this end, we (1) inferred whole-paranome *K*_S_ distributions, (2) performed co-linearity analysis, which classifies the genomic context of gene duplications as dispersed gene pairs or segmental duplications, and (3) employed a recently proposed probabilistic gene tree reconciliation approach designed to test hypotheses about ancient WGDs and to estimate lineage-specific duplication and loss rates. In contrast to the recently published study on this topic [18], we find little support for an important role of WGDs during hexapod evolution. Alternatively, we propose that mainly small-scale gene duplication, together with instances of segmental duplication, possibly mediated through homologous recombination guided by surges in transposon activity, explains the observed duplication signal in hexapods.

## Results

### Delineation of paranomes

We selected 20 high-quality, well-annotated genomes from the available hexapod genome sequences to represent the widest taxonomic diversity of this group. We included the genome of the chelicerid *Limulus polyphemus*, where there is compelling evidence for an ancient genome duplication [16], in our analysis as an outgroup. Table S1 lists the accession numbers of assembled genomes, as well as details on sequencing technology and assembly output. Within-genome sequence similarity searches, followed by MCL clustering, were used to detect paralogous gene pairs (see “Methods”), the number of which varied widely among the analyzed hexapod genomes, ranging from 1225 in the collembolan *Holacanthella duospinosa* to 21,073 in the dipteran *Aedes aegypti* (Table S1).

### Synonymous divergence and co-linearity among gene pairs

Gene duplicate age distributions were inferred by estimating the expected number of synonymous substitutions per synonymous site (synonymous distance or *K*_S_) across the paranome following the approach of Vanneste et al. [24] (see “Methods”). Ancient WGDs result in a characteristic pattern of a peak in distributions of gene duplicates of similar age (similar divergence at synonymous sites) that tend to be in co-linear regions within a genome. The genome of *L. polyphemus* showed such a distinct peak of *K*_S_ values around *K*_S_ ≈ 0.8 (Fig. 1b). The light blue violin plot above the *K*_S_ distributions in Fig. 1 represents the distribution of anchor pair *K*_S_ values found in co-linear blocks (drawn in the inlayed circos plots), which again shows an increase in frequency around *K*_S_ ≈ 0.8 in the case of *L. polyphemus* (Fig. 1b). This pattern is consistent with the ancient WGD reported for this species [16]. A second peak for the more ancient WGD event hypothesized for *L. polyphemus* is not observed in neither the whole-paranome nor anchor pair *K*_S_ distribution, likely due to the age of this event exceeding the window for which *K*_S_ distributions can be used for WGD detection [21, 25].

**Fig. 2:**
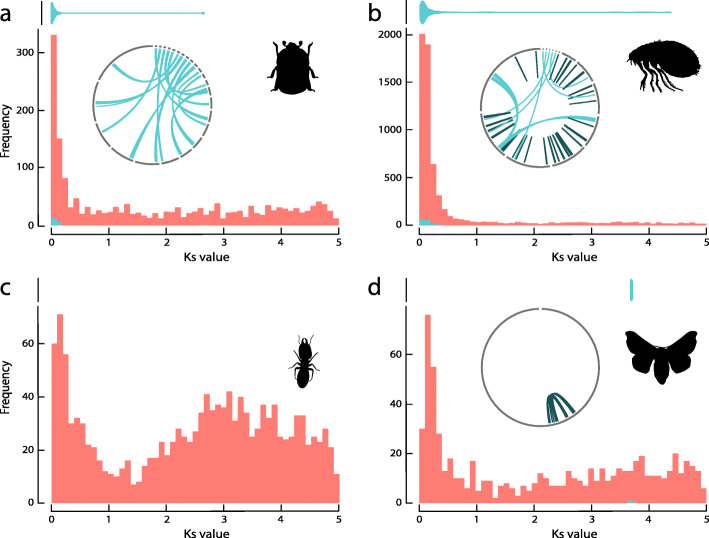
*Ks* frequency distributions and circos plots of co-linear blocks for gene duplicates in the genomes of **a***Aethina tumida*, **b***Ctenocephalides felis*, **c***Zootermopsis nevadensis*, and **d***Bombyx mori*. Color annotation is as in Fig. 1. The silhouette of *C. felis* was derived from freepik.com, while the silhouette from *Z. nevadensis* was derived from cannypic.com

By contrast, none of the hexapod genomes in our study showed a similar pattern consistent with ancient WGD. Instead, we observed several different patterns of gene duplication among hexapod genomes (Figs. 1d and 2, and Figure S1). Most of the genomes in this study showed a high number of young duplicate pairs (low *K*_S_, Figure S1), resulting in the L-shaped distribution that is characteristic for the continuous process of small-scale gene duplication (SSD) and loss [21, 34]. Representative patterns were, for instance, observed in the genomes of *Aethina tumida* and *Ctenocephalides felis* (Fig. 2). The collembolan hexapod *Folsomia candida* exhibited relatively high numbers of duplicates (over 20,000 duplicate pairs, Table S1) and has been assumed to have undergone a lineage-specific WGD in a previous study [18]. If true, we hypothesized that a distinct peak in the whole-paranome *K*_S_ distribution should be observed that coincides with an increase of anchor gene pairs in the same *K*_S_ range, as for instance in the case of *L. polyphemus* (Fig. 1c). Instead, in *F. candida* only a gradual decline in the number of duplication events for increasing *K*_S_ was observed, rather than a distinct peak. Although the *F. candida* genome did, indeed, contain a substantial number of co-linear blocks (55, Table S1), the distribution of anchor pair *K*_S_ values in these co-linear blocks exhibited a similar distribution with declining density for larger *K*_S_ values (blue violin plot Fig. 1d). This pattern suggests that the emergence and loss of such co-linear blocks is a continuous process, reminiscent of small-scale gene duplication (Fig. 1d, *K*_S_ histogram). The reason for this atypical shape of the collembolan anchor pair *K*_S_ distribution needs further investigation, but based on co-linearity analysis (as well as macrosynteny and phylogenomic analyses, see further), we see no reason to invoke a WGD event.

Similar observations were made for other hexapods: none of these genomes retained duplicates co-linearly organized in the way expected under WGD. As a matter of fact, in all hexapod species, most gene pairs were classified as dispersed duplicates not being physically linked in co-linear blocks (Table S1, Fig. 2, Figure S1). Co-linear segments were observed in six out of the 20 genomes (Fig. 2, Figure S1), but in most genomes, these segmental duplications were present in low numbers (Table S1). Larger numbers of duplicated co-linear segments, such as described above for *F. candida*, were also found in the genomes of *A. tumida* and *C. felis* (Table S1, Figure S1). However, similar to the pattern in *F. candida*, in these species the gene pairs organized in co-linear segments were recent and did not coincide with any peaks in *K*_S_ age distributions (Figs. 1d and 2a, b). In *B. mori*, a recent study [18] reported 728 syntenic chains of which 83 could potentially represent segmental duplications. In contrast, we only detected two co-linear segments (consisting of six and seven gene pairs, respectively, Fig. 2d), which is substantially less. To verify whether our more stringent parameter settings in MCScanX could explain the large difference in number of segmental duplications retrieved, we first increased the *E*-value cutoff for all-against-all BLASTP searches with *B. mori* proteins from 10e−10 to 10e−5 (as applied by the Li et al. study [18]). This caused an increase of the number of co-linear genes from 13 to 94. We then used three genes to seed a co-linear block and applied a Manhattan distance of 40 in our MCScanX analysis. This resulted in the identification of 10 co-linear blocks, consisting of 94 co-linear gene pairs, which is still 8 times less than the number of co-linear blocks identified previously [18]. Further decreasing the stringency of these parameter settings eventually yielded 13 co-linear blocks, still a much lower number than previously reported [18]. Changing these parameter settings with regard to the analysis of the *L. polyphemus* genome resulted in an increase from 7 co-linear blocks with 44 gene pairs, to 14 co-linear blocks with 79 gene pairs. The *K*_S_ values of these gene pairs fall within the range of detected *K*_S_ peak of *K*_S_ ≈ 0.8 (Fig. 1b), which is in line with WGD in this species.

### Genome structure of segmental duplications and macrosynteny patterns

Long co-linear blocks of paralogs covering large fractions of the genome are usually considered to support WGD events. In the case of fragmented genome assemblies, genuine WGD-derived co-linear blocks most probably reside on different scaffolds (Fig. 1a). Indeed, the previously inferred ancient WGD event in the chelicerid *L. polyphemus* shows exactly this pattern (Fig. 1b). Of the hexapods we studied, *A. tumida* is the only genome with substantial numbers of co-linear segments located on different scaffolds (Fig. 2a). However, a spurious pattern similar to this can also arise if excessive allelic variation in the genome assembly prevents the collapse of haplotigs into single contigs. Therefore, we tested this explanation by analyzing sequence read coverage of contigs, expecting a drop in coverage among two contigs if they are two haplotigs covering the same genomic region. Indeed, we found that the mean sequence read coverage for *A. tumida* is 124.03× with a 95% confidence interval (CI) between 123.8 and 124.5 assuming normal distribution (Figure S2A). By contrast, the coverage of the co-linear segments is on average 88.82× (95% CI 64.6–119.9, Figure S2B), which is below the lower bound of the 95% CI of coverage among 1000 random contigs. Thus, we conclude that at least some of these co-linear blocks may in fact correspond to haplotigs that did not collapse into one scaffold, and do not correspond to bona fide duplicated regions.

In the *C. felis* genome, we inferred 49 co-linear regions, of which 41 are located on the same scaffold (Fig. 2b, inlay circos plot). Thirty-one of these are organized as palindromes, while the remaining 10 are organized as tandem repeats, which do not support WGD as a source of gene duplication in this species. Similarly, 14 out of 55 co-linear blocks in *F. candida* are located within the same scaffold (Fig. 1d). Importantly, no significant drop in sequence read coverage in these co-linear blocks was found, suggesting that these represent true segmental duplication events [35]. Given the low *K*_S_ values of anchor gene duplicates in *F. candida*’s co-linear blocks (blue violin plot in Fig. 1d, see section above), we infer that they evolved recently. However, if these co-linear blocks would have emerged as a result of a recent WGD event, most should still reside on different chromosomes. By contrast, we observe 14 co-linear blocks to reside within the same chromosome, which does not support WGD as a source of gene duplication in this species. In the case of *B. mori* (where a WGD event was proposed in its lepidopteran ancestor), the two co-linear blocks were also located on a single scaffold (Fig. 2d).

Extensive gene loss and genomic rearrangements may, however, cause substantial “erosion” of co-linear patterns through evolutionary time. Nevertheless, in the absence of conserved gene order, the chromosome-scale distribution of gene duplicates is still expected to be conserved to more or lesser extent, a pattern referred to as macrosynteny [28]. Following Nakatani and McLysaght [23], we further visualized the position of putative gene duplicates in a scatter plot representation for all species where at least one co-linear segment was found (Table S1; Figure S3). Although this visualization can be challenging due to the fragmented nature of some of the included assemblies, these plots clearly indicate that large-scale patterns of within-genome synteny are lacking in the examined genomes. A notable exception is *F. candida*, where peculiar non-random patterns can be observed (Figure S3G). However, the observed organization of gene duplicates for this species is not compatible with WGD either. Recently, we showed that at least some of *F. candida*’s segmental duplications are highly enriched in transposons [35], suggesting transposon-mediated gene duplication or TE proliferation. This remains however speculative and is subject of ongoing research. Taken together, the absence of both substantial co-linear segments and large-scale syntenic patterns suggests no role for WGD in the evolution of hexapods, at least in the evolutionary time frame that allows the inference of WGD events from genome structure.

### Phylogenomic gene tree-species tree reconciliation

We conducted phylogenomic analyses to further investigate patterns of gene duplication and loss, and potential ancestral large-scale duplication events, in a phylogenetic context. To this end, we applied a recently developed Bayesian gene tree reconciliation approach to estimate parameters of a stochastic model of gene family evolution that accounts for duplication, loss, and WGD events, while considering uncertainty in the gene trees by employing amalgamated likelihood estimation (ALE) [22]. In a gene tree reconciliation approach towards WGD inference, we seek to explain the evolution of a set of gene family trees in the context of a known species tree in terms of a set of evolutionary events, in our case, gene duplication and loss events. Most approaches do not use a model of gene family evolution and perform some flavor of parsimony-based reconciliation, effectively counting for each branch in the species tree the number of gene tree clades with a least common ancestor (LCA) that corresponds to that branch in the species tree, among some set of eligible gene tree clades (for instance focusing only on clades with high bootstrap support values in the gene tree or some other subset defined by a filtering criterion). There are several potential problems with such naive reconciliation approaches, among which the reliance on a single estimated gene tree topology is perhaps the most obvious one. Also the assumption that the LCA reconciliation is the true reconciliation can be troubling, as at least one study reported that the most parsimonious reconciliation differs from the true reconciliation in about 19% of the examined cases [36].

In the context of WGD inference, another issue is when to decide whether a given number of inferred duplication events on a particular branch is sufficiently high to infer a polyploidization event. The approach taken by Li et al. [18] is to estimate a background duplication and loss rate for the entire data set using WGDgc [37], and use this estimated rate to simulate a set of gene trees with and without WGD. Based on these simulations, they perform a test to decide whether a particular observed number of duplicates are significantly higher than their simulations without WGD, and not significantly less than their positive simulations with WGD. There are however again several potential issues with such an approach. In particular, the assumption of constant duplication and loss rates across lineages that underlies the simulation procedure has been shown to be inaccurate in the context of WGD inference [22]. In the Bayesian approach of Zwaenepoel and Van de Peer [22], a model of gene family evolution that includes the background duplication and loss process and WGD is used to perform gene tree reconciliation directly in a model-based framework, alleviating the need for potentially problematic simulation schemes. Furthermore, in the Bayesian approach, we can account—in a systematic way—for variation in gene duplication and loss rates across the species tree. We note that with the approach we take here, as with any method that does not take genome structure into account, we cannot distinguish between a WGD and an episodic burst of small-scale or segmental gene duplication events.

**Fig. 3:**
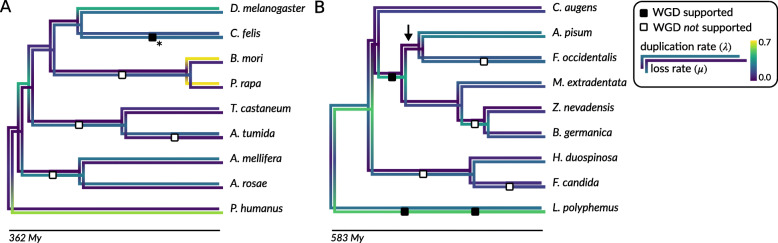
Branch-wise marginal posterior mean duplication (upper branches) and loss rates (lower branches) for the two nine-taxon species trees. **a** Holometabola tree, **b** Hemimetabola (and others) tree. The arrow in **b** indicates the branch where the root of the Holometabola tree in **a** connects to the tree in **b**. The hypothetical WGDs considered in the Whale analyses are indicated along the branches by rectangular boxes, where black indicates WGD hypotheses with a significantly non-zero retention rate (i.e., there is statistical support for a significant deviation from the background duplication-loss process, thus providing support for a proposed WGD hypothesis) and white boxes indicate non-supported WGD hypotheses (i.e., the retention rate does not differ significantly from 0). Please note the caveat with regard to the *C. felis* WGD hypothesis (indicated with an asterisk) as indicated in the main text

For the sake of computational tractability, we considered two trees of 9 species, the first comprising the Holometabola with outgroup *Pediculus humanus* and the second representing the other hexapod groups included in this study with outgroup *L. polyphemus*. We marked 11 hypothetical WGDs on these trees, based on both the co-linearity analyses in this study and the results of Li et al. [18]. Using the simplest possible model, assuming constant rates across the species tree, we found the posterior mean duplication rate (*λ*) across the Holometabola tree to be 0.00215 (events/gene lineage/My) with 95% of the posterior density in [0.00212, 0.00217]. Similarly, a loss rate (*μ*) of 0.00146 [0.00143, 0.00150] was obtained. Employing a branch-wise rates model with an independent rates (IR) prior (see “Methods”) indicated increased duplication rates in Lepidoptera, and an increased loss rate in the branch leading to *P. humanus* (although, in general, estimated rates on isolated branches near the root should be interpreted with caution [22] (Fig. 3a). For the branch leading to the model species *Drosophila melanogaster*, we estimated a duplication rate of 0.00427 [0.00416, 0.00438] and loss rate of 0.00248 [0.00236, 0.00261] events/gene lineage/My, estimates which are well in accord with previous estimates for *D. melanogaster* (e.g., *λ* = 0.0023 [34], *λ* = 0.0050 [38]). We do not find support for WGD in the ancestor of the Diptera, as previously reported, and our analyses could not confirm recently suggested putative WGDs in the stem branches of Lepidoptera, Coleoptera, and Hymenoptera [18]. Performing an analysis with a strongly informative prior on the duplication and loss rates, assuming little rate heterogeneity across branches of the species tree—a model more akin to the simulations employed by Li et al. [18]*—*did not support any of these hypothetical WGDs. Of all WGD hypotheses indicated along this species tree, only a putative *C. felis* event received significant support with a decisively non-zero retention rate (*q*) in all analyses (Figure S6). We note that an inspection of three MCMC chains revealed in one of the chains a second mode in the posterior distribution with *q* ≈ 0 and an increased duplication rate for this branch (Figure S7). However, all chains eventually converge on the same distribution (Figure S7), which likely represents a dominating mode in the posterior where the vast majority of posterior mass is located. Nevertheless, this suggests that the posterior distribution could be multimodal, with besides this dominating mode, the possibility of multiple small “peaks” separated by large “valleys” of low posterior probability, and that the MCMC algorithm may have trouble crossing these valleys. We further note for this putative event in *C. felis* that reconciled trees sampled from the posterior revealed that a very large number of duplication events were reconciled to the hypothetical WGD node, but each of which with very low posterior probability (Figure S8). Furthermore, the *K*_*S*_ distribution for this species shows much more recent duplicates than any of the other examined genomes, but does not show a peak in the distribution nor co-linear blocks indicative of WGD. Together, this suggests that the non-zero retention rate for this branch is spurious and that the signal in the gene trees mistaken for a WGD event more likely corresponds to an increased gene duplication rate due to some other mechanism.

For the other species tree (Fig. 3b), analysis under the constant-rates model revealed a tree-wide duplication rate of 0.00160 [0.00158, 0.00161] and loss rate of 0.00195 [0.00192, 0.00198] events/gene lineage/My. Bayesian inference with Whale using models of branch-wise duplication and loss rate variation was more challenging for this tree, and convergence was not attained for the full branch-wise rate models, presumably due to the long outgroup branch that included hypothetical WGDs. To mitigate these issues, we constrained the two branches stemming from the root to have identical rates. Under the IR model, we obtained duplication and loss rates within a similar range as for the Holometabola tree (Fig. 3b). Posterior inferences under an autocorrelated prior were quite different from results for the IR prior, but did not result in any qualitative differences with respect to potential WGDs. In both cases, the data showed to be compatible with a previously suggested putative Insecta-shared WGD [18] (*q* = 31% [29%, 33%] for the IR model), although the retention rate was highly sensitive to the prior used (Figure S11). The hypothesized WGDs in *L. polyphemus* were also recovered with high retention rates, yet with a very high posterior variance (94% [77%, 100%] for the oldest WGD and 44% [32%, 58%] for the most recent putative WGD). This high variability is not surprising as this branch represents almost 600 My of evolution, and the only temporal information in the ALE-based approach used in Whale comes from tree topologies and not branch lengths per se. In line with the *K*_S_ distribution and co-linearity analyses in the present study, we do not find compelling evidence for large-scale duplication events in the stem of Dictyoptera (cockroaches, termites, and mantises), stem of Colembolla (springtails), the branch leading to *Frankliniella*, or the branch leading to *Folsomia* (Figure S11).

## Discussion

Gene duplication has been appreciated as an important factor in evolution for a long time [1, 39]. Our current study based on the analysis of whole-paranome *K*_S_ distributions, intragenomic co-linearity, and gene tree reconciliation for 20 high-quality hexapod genomes confirms gene duplicate abundance in this taxonomic group, with estimated rates of small-scale duplication and loss on the order of 0.002 events/gene/My. As expected, most duplicate gene pairs in hexapods are of recent origin, compatible with the continuous process of small-scale duplication and loss. However, some hexapod genomes, such as those of collembolans, show substantial retention of ancient gene duplicates, suggesting variation in the rates of the continuous duplication and loss process across the hexapod phylogeny.

Our current study does not provide evidence for multiple whole-genome duplication events to have occurred during the evolution of hexapods. We unveiled a few segmental or large-scale duplications, but only in some of the genomes, and mostly of recent origin. The fact that these duplications were often located on the same scaffold is difficult to reconcile with WGDs, especially for relatively recent hypothesized WGDs. For example, *F. candida* and *C. felis* contained within-genome co-linear regions, which did not coincide with alternative peaks in *K*_*S*_ distributions, and of which a substantial fraction was intra-chromosomally distributed (Figs. 1d and 2b). Some intra-chromosomal co-linearity is expected to arise during rediploidization, a process associated with chromosomal rearrangements such as chromosomal fusions and translocations [40–42]. However, on average, the majority of co-linear blocks arising from WGD should reside on different chromosomes [23, 26–28], especially in case of recent WGD events. Therefore, the high frequency of recently evolved intra-chromosomal co-linearity observed in *C. felis* and *F. candida* is not in concordance with a WGD scenario. Large-scale macrosynteny pattern did not suggest a role for WGD in the evolution of hexapod genome structure either.

Identifying co-linearity is not straightforward, particularly in the case of ancient duplications where subsequent genome dynamics and restructuring may have erased the co-linearity signature to a large extent [22]. Also, the robustness of such analysis is dependent on assembly quality and applied analysis tool [43]. In this respect, the case of *B. mori* is interesting because this genome is of high quality and was analyzed using three independent co-linearity analysis tools [18, 23]. Li et al. [18] identified 728 potentially syntenic chains using the SynMap tool from the CoGe platform [44] that included 2210 genes, and suggested that 83 chains were associated with an ancient WGD event in Lepidoptera identified by their MultitAxon Paleopolyploidy Search (MAPS) algorithm [18]. More recently, Nakatani and McLysaght [23] visualized the position of these chained BlastP hits from the Li et al. analysis [18] on the silkworm chromosomes. They found that the majority of these duplicates were not chained, but were randomly distributed over the entire genome instead of organized in syntenic blocks. As mentioned above, extensive chromosomal rearrangements following WGD may have randomized paralog distribution throughout the *B. mori* genome over evolutionary time. This explanation seems unlikely, however, given the high levels of macrosynteny that was observed between *B. mori* and the coleopteran genome of *Tribolium castaneum* [23]. In the current study, we used MCScanX with conservative parameter settings and identified only two true segmental duplications, which were organized on one scaffold (Fig. 2d). Adjusting the parameter settings to the ones used by Li et al. [18] did not retrieve comparable results. Taken together, our independent analysis confirmed the previous argument that WGD did not contribute to genome evolution in *B. mori* [23]. This conclusion is corroborated by our gene tree-species tree reconciliation analysis, which did not find support for a putative WGD in the lepidopteran stem branch.

Phylogenomic gene tree reconciliation analyses provided further insights into the phylogenetic patterns of gene duplication and loss across hexapods, as well as more ancient hypothetical large-scale duplication events. Duplication and loss rates varied across lineages, but remained within the same order of magnitude across the entire phylogeny. Using the statistical approach implemented in Whale to assess putative WGDs along those branches that were previously investigated [18], we failed to confirm the conclusions of this study in all but one case. Despite the fact that our taxon sampling was more limited, these results cast doubts on the methodology of the previous study and perhaps the suitability of transcriptomic data to infer gene family evolutionary processes. In particular, the assumption of constant rates across lineages, as applied in the previous hexapod WGD study [18], can seriously compromise inference of WGDs [22]. Conclusively refuting a hypothesized ancient WGD event is of course challenging, but model-based statistical inference can indicate under which assumptions what conclusions are acceptable. We showed that if we assumed the rate of gene duplication and loss to vary across lineages, i.e., duplication and loss, follow independent relaxed molecular clocks, gene trees of multi-copy gene families did not provide support for all but one of the entertained WGD hypotheses. We stipulate that to further substantiate these results, an increased taxon sampling remains desirable, breaking up long branches for which a WGD is hypothesized.

Our failure to confirm putative WGD events in hexapods seems also supported by Hox gene cluster organization. As mentioned previously, the *L. polyphemus* genome contains up to four copies of each Hox gene, supporting the hypothesis that this genome evolved through two rounds of WGD [16]. A literature survey among several published hexapod genomes (*D. melanogaster*, *T. castaneum*, *F. candida*, *Orchesella cincta*, *Acyrthosiphon pisum*, Z*ootermopsis nevadensis*) showed that these genes are represented in single copy [20, 45–48], which is in line with our current findings. While Hox gene clusters are very tightly organized as one dense gene cluster in vertebrate genomes, hexapod Hox gene clusters seem to show a more differential pattern of gene dispersion, often interspersed by other open reading frames and long stretches of non-coding DNA [35, 45, 49].

An alternative scenario that would produce the observed signatures of large-scale gene duplication events in our and previous studies [18] are bursts of transposable element (TE) activity [30]. For example, duplication-dependent strand annealing was elucidated as the mechanism explaining their formation in the *D. melanogaster* genome [50]. Hotspots of TEs cause an increase in homologous regions, providing more opportunity for homologous recombination and unequal crossing over to drive gene amplification [51]. In such case, segmental duplications reside in genomic regions with high TE activity/abundance [52]. A recent systematic survey of TE activity across insect genomes provided evidence for ancient bursts of TE activity [53] in many insect species, which coincide with the *K*_S_ distribution of gene pairs detected in this study. For instance, *Z. nevadensis* showed an increased frequency of gene duplicates with *K*_S_ values between 2 and 4 (Fig. 2c). This coincides with a second broad peak of LINE transposon abundance in the Petersen study [53]. Similar concordant patterns between *K*_S_ distributions of gene duplicates and divergence distributions of the DNA transposon, LTR transposon, and rolling circle transposon families [53] were observed in *P. humanus* and *Apis mellifera*. Moreover, co-linear gene blocks in the genome of *F. candida* were found to be spatially associated with high densities of transposable element [35], suggesting a link between transposon activity and segmental duplication in the evolution of this genome. Finally, evidence was found for the involvement of high-density transposon regions facilitating gene family expansion of odorant receptors in ants [35]. Based on these observations, a tentative hypothesis emerges that bursts in transposon activity early during the evolution of some hexapod lineages may provide the basis for segmental duplication, by facilitating duplication-dependent strand annealing as main mechanism of gene duplication (Fig. 1c). In order to test this hypothesis, junctions of co-linear blocks could be examined for particular transposon sequence features. Such analysis was performed within the human genome, where enrichment of Alu short interspersed element (SINE) sequences near or within such junction was observed [51]. Moreover, assessing coincidence of our current age estimation based on *K*_S_ distributions with estimations of transposition rates in the evolutionary past could provide further support for our hypothesis. Historical transposition events could be dated in a phylogenetic context, as was shown for past transposition rate estimations of pogo-like transposable elements in different *Fusarium* species [54]. However, we are currently unaware of a method to reliably calibrate these two time indications against each other.

## Conclusions

The analysis of intragenomic co-linearity, *K*_S_ distributions, and gene tree-species tree reconciliation across a wide taxonomic range of hexapod genome sequences suggests that gene duplication is pervasive among hexapods and that species differ in the degree to which ancient gene duplicates have been retained. Interpreting our results in the light of recent studies, we speculate that TE activity might explain the observed patterns of bursts of gene duplication, while compelling evidence for an important role of WGD in hexapod evolution is missing.

## Methods

### Data sampling

Sample selection was guided by the availability of high-quality assembled genome sequences and taxonomic breadth. We aimed for a balanced distribution of genomes over hexapod diversity, and selected two genomes for each hexapod order, if available. In case multiple genomes were available for a given hexapod order, we selected the two most contiguous ones. In total, 20 high-quality hexapod genomes were compiled. The genome of the non-hexapod *Limulus polyphemus* was included as an exemplary lineage where WGD is well described [16]. Supplementary Table 1 lists sample names with associated accession numbers of assembled genomes, as well as details on sequencing technology and assembly output.

### *K*_S_ distribution analysis

For paralogous gene families of two or more members, we estimated the expected number of synonymous substitutions per synonymous site (*K*_S_ value) for each node in the gene family tree using the approach of Vanneste et al. [24] as implemented in the “wgd” pipeline [55]. In brief, for each species, an all-against-all protein similarity search was done using BlastP with an *e*-value cutoff of 1e−10. A sequence similarity graph was constructed and gene families were inferred using Markov Clustering with MCL (inflation factor = 2.0) [56]. The amino acid sequences of gene families were used to infer a multiple sequence alignment with MAFFT v7 (using options: “--amino –maxiterate 1000 –localpair”) [57]. This alignment was then used as a guide for obtaining a gap-stripped codon-level alignment. For each gene family, pairwise *K*_S_ values were estimated through maximum likelihood using CODEML (with runmode = − 2) from the PAML package [58], using the Goldman & Yang model (GY94) and the F3x4 method for estimating equilibrium codon frequencies. For each family, an approximate phylogenetic tree was obtained using FastTree with default settings [59], which was then used to construct node-weighted empirical *K*_S_ distributions. The final set of *K*_S_ values for each genome is represented as a weighted histogram, where the *y*-axis represents the number of duplication events (not duplicate pairs)*,* to detect temporal patterns of gene duplication.

### Co-linearity analysis

To detect co-linear blocks, we used the Multiple Co-linearity Scan toolkit (MCScanX), using standard settings [60], except where indicated. The duplicate gene classifier within MCScanX uses the MCScan algorithm to classify the genomic context of gene duplications into three groups: segmental (including putative WGD-derived duplicates), tandem, or dispersed. Initially, all genes classify as singletons, while all gene pairs identified by BlastP are assigned dispersed duplicates. The segmental/WGD label is assigned to anchor pairs in intragenomic co-linear blocks [60]. Genome-wide co-linearity was visualized using Circos [61].

#### Probabilistic gene tree-species tree reconciliation

We used the recently developed Whale approach for statistical assessment of WGD hypotheses [22]. For the analyses using Whale (v0.3), we considered two species trees of nine species, the first comprising the Holometabola with as outgroup *P. humanus*, and the second representing the other hexapod groups included in this study with as outgroup *L. polyphemus.* Species trees with branch lengths in calendar time were obtained from the TimeTree database [62]. We assumed 11 WGD hypotheses on these trees, informed by both the co-linearity analyses in this study and the results of Li et al. [18]. For both sets of species, we inferred gene families using OrthoFinder v2.3.3 [63]. To rule out de novo origin of gene families in arbitrary subtrees of the relevant species tree, we filtered out gene families that did not contain at least one gene in both species’ clades stemming from the root in the associated species tree. We further filtered out large gene families according to a Poisson outlier criterion. Specifically, under the assumption that the total family size *X* across species is approximately Poisson distributed, we have that *Y* = *2√X* ∼ Normal (median(*Y*), 1). Based on this assumption, we filtered out all gene families for which *Y* > median(*Y*) + 3. This resulted in 6937 gene families for the Holometabola set and 6712 gene families for the other species set. Pre-alignment masking of putatively non-homologous characters was performed using Prequal v1.01 [64], after which a protein multiple sequence alignment (MSA) was inferred for each gene family using MAFFT v7 [57]. For each alignment, a sample from the posterior distribution of phylogenetic trees was obtained using MrBayes v3.2.6 [65] using the LG+Γ4 substitution model and default exponential priors on the branch lengths. We ran the MCMC for 110,000 generations for each family, recording a sample every 10 generations after discarding the first 10,000 generations as burn-in. The conditional clade distribution (CCD) was computed using the ALEobserve tool from the ALE suite v0.4 [66].

We performed Bayesian inference under the DL+WGD model in Whale using different priors and model structures for the duplication and loss rates across lineages of the species tree. For both species sets, we initially performed an analysis assuming constant rates of duplication and loss across the species tree and no WGD hypotheses. We assumed an exponential prior distribution with mean 0.005 events per gene lineage per million year (ev/lineage/My) on both the duplication and loss rate and a Beta (10, 2) hyper prior on the *η* parameter of the geometric prior distribution on the number of lineages in a gene family present at the root of the species tree. We used the marginal posterior mean *η* value from the constant-rates analysis for the relevant species set in all subsequent analyses for the same species set. We next performed an analysis using hierarchical independent branch-wise rates prior, where duplication and loss rate across branches are assumed to be independent and identically distributed following a log-normal distribution. We assumed an exponential distribution (with mean 0.5) on the mean duplication and loss rate and an InverseGamma (5, 1) prior on the variance of the log-normal distribution. We used uniform priors on the retention rates for all WGDs. Last, we performed Bayesian inference using the geometric Brownian motion (GBM) prior with strong assumed phylogenetic correlation (*ν* = 0.1), to investigate the influence of different assumptions on the branch-wise duplication and loss rates on the estimated WGD retention rates. For the analyses under the GBM prior, we used the same priors on the mean duplication and loss rates and retention rates. Throughout our analyses of the second (non-Holometabola) tree, convergence issues in the MCMC required us to constrain the duplication and loss rates to be identical for the two branches stemming from the root. Throughout our study, we use multiple pilot runs for subsets of 1000 gene families to investigate convergence and base our reported estimates on a chain ran for 11,000 generations with a burn-in of 1000 generations for the full data set. Analysis for the full data set is very computationally intensive, and therefore, we compare the obtained MCMC chains for the full data set with multiple chains for the random 1000 gene family subsets visually to assess convergence (e.g., Figures S1, S2).

### Sequence read coverage analysis

To test whether co-linear regions in *A. tumida* show a drop in sequence read coverage, we used bedtools genomecov package version 2.28 to calculate the coverage of each basepair in the *A. tumida* genome. Subsequently, mean coverage over co-linear blocks was calculated and compared to the mean coverage of 1000 randomly sampled genome regions of the same length as the co-linear blocks including a 95% confidence interval.

## Supplementary information

**Additional file 1: Figure S1.** Ks frequency distribution graphs and circos plots of collinear syntenic blocks for gene duplicates in the genomes of A *Aedis aegypti*, B *Acyrthosiphon pisum*, C *Apis mellifera*, D *Athalia rosae*, E *Bemisia tabaci*, F *Blattella germanica*, G *Campodea augens*, H *Drosophila melanogaster*, I *Frankliniella occidentalis*, J *Holacanthella duospinosa*, K *Medauroidea extradentata*, L *Orchesella cincta*, M *Pediculus humanus*, N *Pieris rapae*, O *Tribolium castaneum*. Orange-red, frequency distribution of gene duplicate bins with identical Ks values; light blue, WGD/segmental duplication event predicted by MSscanX; inlay, circos plot co-linear blocks. **Figure S2.** Histograms of sequence read coverage distribution (bins of 20 counts) among scaffolds of *Aethina tumida’s* genome assembly: 1000 random contigs (A) and contigs with co-linear regions (B). **Figure S3.** Scatter plots of putative gene duplicates (BlastP hits with e-value < 10–10) for species that contain at least one segmental duplication. A) *L. polyphemus*, b) *A. aegypti* c) *A. tumida*, d) *B. germanica*, e) *B. mori*, f) *C. felis*, g) *F. candida*. Co-linear blocks identified by MCScanX are indicated as red dots. Scale on horizontal axis in bp. **Figure S4.** Trace plots for the MCMC samples for the Holometabola data set with the IR prior. In black results for the full data set are shown (10,000 generations after 1000 generations as burn-in, showing every iterate), whereas the other transparent colors show three replicate chains for a random subset of 1000 gene families (20,000 generations after 1000 as burn-in, showing every second iterate). Duplication (λ) and loss (μ) rates are shown on a log10 scale, and subscripts denote branches of the species tree. **Figure S5.** Marginal posterior distributions for the MCMC samples for the Holometabola data set with the IR prior. Interpretation is as in Figure S4, but here we show the rates on the original scale. **Figure S6.** Marginal posterior distributions for retention rates (*q*) of the five hypothetical WGD events marked along the Holometabola tree. The upper row shows results under the IR prior, whereas the lower row corresponds to results under the GBM (autocorrelated rates) prior (see methods). Note that the distributions under the GBM prior for the Lepidoptera, Coleoptera and Hymenoptera events are vanishingly small but are shown on the same scale as the upper row for the sake of comparison. **Figure S7.** A distinct mode for the parameters associated with the *C. felis* branch was observed in one of the chains under the IR prior for the Holometabola tree, indicating the possible problem of inefficient sampling of multimodal distributions in Whale. Results from three independent chains are shown in blue, orange and green respectively. (a & d) Marginal posterior distributions for the duplication (λ) and retention (q) rate associated with the *C. felis* branch for two chains. (b,c & e) Trace plots for duplication, 2 loss (μ) and retention rates associated with the *C. felis* branch for the same two chains. (f) Trace plot of the log likelihood for these chains. **Figure S8.** Posterior reconciliation probabilities of gene duplicates reconciled to the hypothetical *C. felis* (A) or Insecta (B) WGDs. The posterior reconciliation probability is calculated as the fraction that a particular clade is reconciled to the WGD node of interest in 1000 reconciled trees sampled from the posterior. Boxplots show the same data but grouped by clade size, showing for the *C. felis* WGD hypothesis a slight trend towards lower reconciliation probabilities for larger clades, whereas this trend is not observed for the putative Insecta event. **Figure S9.** Trace plots for the MCMC samples for the non-Holometabola data set with the IR prior. In black results for the full data set are shown (10000 generations after 1000 generations as burn-in, showing every iterate), whereas the other transparent colors show three replicate chains for a random subset of 1000 gene families (20000 generations after 1000 as burn-in, showing every second iterate). Duplication (λ) and loss (μ) rates are shown on a log10 scale and subscripts denote branches of the species tree. **Figure S10.** Marginal posterior distributions for the MCMC samples for the non-Holometabola data set with the IR prior. Interpretation is as in Figure S8 but here we show the rates on the original scale. **Figure S11.** Marginal posterior distributions for retention rates (*q*) of the seven hypothetical WGD events marked along the non-Holometabola tree. The upper row shows results under the IR prior, whereas the lower row corresponds to results under the GBM (autocorrelated rates) prior (see methods). Note that the distributions under the GBM prior for the Colembolla and Polyneoptera events are vanishingly small but are shown on the same scale as the upper row for the sake of comparison. **Table S1.** General specifications of species included in this study. Gene pairs, the number of gene pairs per hexapod species used as input for Ks calculation, co-linearity analysis and gene tree-species tree reconciliation analysis. Gene pairs with Ks values of 0 and higher than 5 were filtered out.

## Data Availability

The datasets analyzed during the current study are available in the National Center for Biotechnology Information (NCBI) repository, ncbi.nlm.nih.gov/genome. Genome sequences of blattella, holacanthella, and medauroidea are available from the i5K initiative and can be downloaded from https://i5k.nal.usda.gov. The genome sequence of *Campodea augens* is published in BioRxiv [67] and was kindly provided by the authors. The following accession numbers were used: *A. pisum* GCF_000142985.2 [48], *A. aegypti* GCF_002204515.2 [68], *A. tumida* GCF_001937115.1 [69], *A. mellifera* GCF_000002195.4 [70], *Athalia rosae* GCF_000344095.1 [71], *Bemisia tabaci* GCF_001854935.1 [72], *Blatella germanica* GCA_000762945.2 [46], *B. mori* GCF_000151625.1 [73], *C. augens* campodea_augens_genome_v1.0 [67], *C. felis* GCF_003426905.1 [74], *D. melanogaster* GCF_000001215.4 [75], *F. candida* fcand_genome.fa (Collembolomics.nl) [35], *Frankliniella occidentalis* GCF_000697945.2 [76], *H. duospinosa* GCA_002738285.1 [77], *Medauroidea extradentata* GCA_003012365.1 [78], *O. cincta* ocinc_genome.fa (Collembolomics.nl) [79], *P. humanus* GCF_000006295.1 [80], *Pieris rapae* GCF_001856805.1 [81], *T. castaneum* GCA_000002335.3 [82], *Z. nevadensis* GCA_000696155.1 [47], *L. polyphemus* GCF_000517525.1 [83].

## References

[1] Ohno S, Wolf U, Atkin NB (1967). Evolution from fish to mammals by gene duplication. Hereditas.

[2] Zhang J (2003). Evolution by gene duplication: an update. Trends Ecol Evol.

[3] De Smet R, Sabaghian E, Li Z, Saeys Y, Van de Peer Y (2017). Coordinated functional divergence of genes after genome duplication in Arabidopsis thaliana. Plant Cell.

[4] Osborn TC, Chris Pires J, Birchler JA, Auger DL, Jeffery Chen Z, Lee H-S (2003). Understanding mechanisms of novel gene expression in polyploids. Trends Genet.

[5] Crow KD, Wagner GP (2006). What is the role of genome duplication in the evolution of complexity and diversity?. Mol Biol Evol.

[6] Yao Y, Carretero-Paulet L, Van de Peer Y (2019). Using digital organisms to study the evolutionary consequences of whole genome duplication and polyploidy. PLoS One.

[7] Landis JB, Soltis DE, Li Z, Marx HE, Barker MS, Tank DC (2018). Impact of whole-genome duplication events on diversification rates in angiosperms. Am J Bot.

[8] Tank DC, Eastman JM, Pennell MW, Soltis PS, Soltis DE, Hinchliff CE (2015). Nested radiations and the pulse of angiosperm diversification: increased diversification rates often follow whole genome duplications. Source New Phytol.

[9] Schranz ME, Mohammadin S, Edger PP (2012). Ancient whole genome duplications, novelty and diversification: the WGD radiation lag-time model. Curr Opin Plant Biol.

[10] Mayrose I, Zhan SH, Rothfels CJ, Magnuson-Ford K, Barker MS, Rieseberg LH (2011). Recently formed polyploid plants diversify at lower rates. Science.

[11] Van de Peer Y, Maere S, Meyer A (2009). The evolutionary significance of ancient genome duplications. Nat Rev Genet.

[12] Mable BK (2004). ‘Why polyploidy is rarer in animals than in plants’: myths and mechanisms. Biol J Linn Soc.

[13] Muller HJ (1925). Why polyploidy is rarer in animals than in plants. Am Nat.

[14] Orr HA (1990). “Why polyploidy is rarer in animals than in plants”; revisited. Am Nat.

[15] Hallinan NM, Lindberg DR (2011). Comparative analysis of chromosome counts infers three Paleopolyploidies in the Mollusca. Genome Biol Evol.

[16] Kenny NJ, Chan KW, Nong W, Qu Z, Maeso I, Yip HY (2016). Ancestral whole-genome duplication in the marine chelicerate horseshoe crabs. Heredity.

[17] Clarke TH, Garb JE, Hayashi CY, Arensburger P, Ayoub NA (2015). Spider transcriptomes identify ancient large-scale gene duplication event potentially important in silk gland evolution. Genome Biol Evol.

[18] Li Z, Tiley GP, Galuska SR, Reardon CR, Kidder TI, Rundell RJ (2018). Multiple large-scale gene and genome duplications during the evolution of hexapods. Proc Natl Acad Sci U S A.

[19] Schwager EE, Sharma PP, Clarke T, Leite DJ, Wierschin T, Pechmann M (2017). The house spider genome reveals an ancient whole-genome duplication during arachnid evolution. BMC Biol.

[20] Leite DJ, McGregor AP (2016). Arthropod evolution and development: recent insights from chelicerates and myriapods. Curr Opin Genet Dev.

[21] Zwaenepoel A, Li Z, Lohaus R, Van de Peer Y (2019). Finding evidence for whole genome duplications: a reappraisal. Mol Plant.

[22] Zwaenepoel A, Van de Peer Y (2019). Inference of ancient whole-genome duplications and the evolution of gene duplication and loss rates. Mol Biol Evol.

[23] Nakatani Y, McLysaght A (2019). Macrosynteny analysis shows the absence of ancient whole-genome duplication in lepidopteran insects. Proc Natl Acad Sci U S A.

[24] Vanneste K, Van de Peer Y, Maere S (2013). Inference of genome duplications from age distributions revisited. Mol Biol Evol.

[25] Tiley GP, Barker MS, Burleigh JG (2018). Assessing the performance of Ks plots for detecting ancient whole genome duplications. Genome Biol Evol.

[26] Jaillon O, Aury J-M, Brunet F, Petit J-L, Stange-Thomann N, Mauceli E (2004). Genome duplication in the teleost fish Tetraodon nigroviridis reveals the early vertebrate proto-karyotype. Nature.

[27] Kellis M, Birren BW, Lander ES (2004). Proof and evolutionary analysis of ancient genome duplication in the yeast Saccharomyces cerevisiae. Nature.

[28] Nakatani Y, McLysaght A (2017). Genomes as documents of evolutionary history: a probabilistic macrosynteny model for the reconstruction of ancestral genomes. Bioinformatics.

[29] Van de Peer Y (2004). Computational approaches to unveiling ancient genome duplications. Nat Rev Genet.

[30] Schrader L, Schmitz J (2019). The impact of transposable elements in adaptive evolution. Mol Ecol.

[31] Jiao Y, Wickett NJ, Ayyampalayam S, Chanderbali AS, Landherr L, Ralph PE (2011). Ancestral polyploidy in seed plants and angiosperms. Nature.

[32] Li Z, Baniaga AE, Sessa EB, Scascitelli M, Graham SW, Rieseberg LH (2015). Early genome duplications in conifers and other seed plants. Sci Adv.

[33] Ruprecht C, Lohaus R, Vanneste K, Mutwil M, Nikoloski Z, Van de Peer Y (2017). Revisiting ancestral polyploidy in plants. Sci Adv.

[34] Lynch M, Conery JS (2000). The evolutionary fate and consequences of duplicate genes. Science.

[35] Faddeeva-Vakhrusheva A, Kraaijeveld K, Derks MFL, Anvar SY, Agamennone V, Suring W (2017). Coping with living in the soil : the genome of the parthenogenetic springtail *Folsomia candida*. BMC Genomics.

[36] Mahmudi O, Sjöstrand J, Sennblad B, Lagergren J (2013). Genome-wide probabilistic reconciliation analysis across vertebrates. BMC Bioinformatics.

[37] Rabier C-E, Ta T, Ané C (2014). Detecting and locating whole genome duplications on a phylogeny: a probabilistic approach. Mol Biol Evol.

[38] Hahn MW, Han MV, Han S-G (2007). Gene family evolution across 12 Drosophila genomes. PLoS Genet.

[39] Taylor JS, Raes J (2004). Duplication and divergence: the evolution of new genes and old ideas. Annu Rev Genet.

[40] Simillion C, Vandepoele K, Van Montagu MCE, Zabeau M, Van de Peer Y (2002). The hidden duplication past of Arabidopsis thaliana. Proc Natl Acad Sci U S A.

[41] Mandáková T, Lysak MA (2018). Post-polyploid diploidization and diversification through dysploid changes. Curr Opin Plant Biol.

[42] Lysak MA, Berr A, Pecinka A, Schmidt R, McBreen K, Schubert I (2006). Mechanisms of chromosome number reduction in Arabidopsis thaliana and related Brassicaceae species. Proc Natl Acad Sci U S A.

[43] Liu D, Hunt M, Tsai IJ (2018). Inferring synteny between genome assemblies: a systematic evaluation. BMC Bioinformatics.

[44] Lyons Eric, Pedersen Brent, Kane Josh, Freeling Michael (2008). The Value of Nonmodel Genomes and an Example Using SynMap Within CoGe to Dissect the Hexaploidy that Predates the Rosids. Tropical Plant Biology.

[45] Pace RM, Grbić M, Nagy LM (2016). Composition and genomic organization of arthropod Hox clusters. Evodevo.

[46] Harrison MC, Jongepier E, Robertson HM, Arning N, Bitard-Feildel T, Chao H (2018). Hemimetabolous genomes reveal molecular basis of termite eusociality. Nat Ecol Evol.

[47] Terrapon N, Li C, Robertson HM, Ji L, Meng X, Booth W (2014). Molecular traces of alternative social organization in a termite genome. Nat Commun.

[48] Richards S, Gibbs RA, Gerardo NM, Moran N, Nakabachi A, Stern D (2010). Genome sequence of the pea aphid Acyrthosiphon pisum. PLoS Biol.

[49] Duboule D (2007). The rise and fall of Hox gene clusters. Development.

[50] Fiston-Lavier A-S, Anxolabehere D, Quesneville H (2007). A model of segmental duplication formation in Drosophila melanogaster. Genome Res.

[51] Bailey JA, Liu G, Eichler EE (2003). An Alu transposition model for the origin and expansion of human segmental duplications. Am J Hum Genet.

[52] Schrader L, Kim JW, Ence D, Zimin A, Klein A, Wyschetzki K, et al. ARTICLE transposable element islands facilitate adaptation to novel environments in an invasive species. Nat Commun. 2014;5 10.1038/ncomms6495. 10.1038/ncomms6495 PMC4284661 25510865

[53] Petersen M, Armisén D, Gibbs RA, Hering L, Khila A, Mayer G (2019). Diversity and evolution of the transposable element repertoire in arthropods with particular reference to insects. BMC Evol Biol.

[54] Le Rouzic A, Payen T, Hua-Van A (2013). Reconstructing the evolutionary history of transposable elements. Genome Biol Evol.

[55] Zwaenepoel A, Van de Peer Y (2019). Wgd—simple command line tools for the analysis of ancient whole-genome duplications. Bioinformatics.

[56] Enright AJ, Van Dongen S, Ouzounis CA (2002). An efficient algorithm for large-scale detection of protein families. Nucleic Acids Res.

[57] Katoh K, Standley DM (2013). MAFFT multiple sequence alignment software version 7: improvements in performance and usability. Mol Biol Evol.

[58] Yang Z (2007). PAML 4: phylogenetic analysis by maximum likelihood. Mol Biol Evol.

[59] Price MN, Dehal PS, Arkin AP (2010). FastTree 2 – approximately maximum-likelihood trees for large alignments. PLoS One.

[60] Wang Y, Tang H, DeBarry JD, Tan X, Li J, Wang X (2012). MCScanX: a toolkit for detection and evolutionary analysis of gene synteny and collinearity. Nucleic Acids Res.

[61] Krzywinski M, Schein J, Birol I, Connors J, Gascoyne R, Horsman D (2009). Circos: an information aesthetic for comparative genomics. Genome Res.

[62] Kumar S, Stecher G, Suleski M, Hedges SB (2017). TimeTree: a resource for timelines, timetrees, and divergence times. Mol Biol Evol.

[63] Emms DM, Kelly S (2015). OrthoFinder: solving fundamental biases in whole genome comparisons dramatically improves orthogroup inference accuracy. Genome Biol.

[64] Whelan S, Irisarri I, Burki F. PREQUAL: detecting non-homologous characters in sets of unaligned homologous sequences. Bioinformatics. 2018; 10.1093/bioinformatics/bty448. 10.1093/bioinformatics/bty448 29868763

[65] Ronquist F, Teslenko M, van der Mark P, Ayres DL, Darling A, Höhna S (2012). MrBayes 3.2: efficient Bayesian phylogenetic inference and model choice across a large model space. Syst Biol.

[66] Szöllősi GJ, Rosikiewicz W, Boussau B, Tannier E, Daubin V (2013). Efficient exploration of the space of reconciled gene trees. Syst Biol.

[67] Manni M, Simao FA, Robertson HM, Gabaglio MA, Waterhouse RM, Misof B, et al. The genome of the blind soil-dwelling and ancestrally wingless dipluran Campodea augens, a key reference hexapod for studying the emergence of insect innovations. Preprint at https://www.biorxiv.org/content/10.1101/585695v3. 10.1101/585695. 10.1093/gbe/evz260 PMC6938034 31778187

[68] Dudchenko O, Batra SS, Omer AD, Nyquist SK, Hoeger M, Durand NC (2017). De novo assembly of the Aedes aegypti genome using Hi-C yields chromosome-length scaffolds. Science.

[69] Evans JD, Mckenna D, Scully E, Cook SC, Dainat B, Egekwu N (2018). Genome of the small hive beetle (Aethina tumida, Coleoptera: Nitidulidae), a worldwide parasite of social bee colonies, provides insights into detoxification and herbivory. Giga Sci.

[70] Weinstock GM, Robinson GE, Gibbs RA, Worley KC, Evans JD, Maleszka R (2006). Insights into social insects from the genome of the honeybee Apis mellifera. Nature.

[71] Mine S, Sumitani M, Aoki F, Hatakeyama M, Suzuki MG (2017). Identification and functional characterization of the sex-determining gene doublesex in the sawfly, Athalia rosae (Hymenoptera: Tenthredinidae). Appl Entomol Zool.

[72] Chen W, Hasegawa DK, Kaur N, Kliot A, Pinheiro PV, Luan J (2016). The draft genome of whitefly Bemisia tabaci MEAM1, a global crop pest, provides novel insights into virus transmission, host adaptation, and insecticide resistance. BMC Biol.

[73] Xia Q, Wang J, Zhou Z, Li R, Fan W, Cheng D (2008). The genome of a lepidopteran model insect, the silkworm Bombyx mori. Insect Biochem Mol Biol.

[74] Driscoll TP, Verhoeve VI, Gillespie JJ, Johnston JS, Guillotte ML, Rennoll-Bankert KE, et al. Cat fleas in flux: rampant gene duplication, genome size plasticity, and paradoxical Wolbachia infection. Preprint at https://www.biorxiv.org/content/10.1101/2020.04.14.038018v1. 10.1101/2020.04.14.038018. 10.1186/s12915-020-00802-7 PMC7305587 32560686

[75] Hoskins RA, Carlson JW, Wan KH, Park S, Mendez I, Galle SE (2015). The release 6 reference sequence of the Drosophila melanogaster genome. Genome Res.

[76] Rotenberg D, Baumann AA, Ben-Mahmoud S, Christiaens O, Dermauw W, Ioannidis P, et al. Genome-enabled insights into the biology of thrips as crop pests. Preprint at https://www.biorxiv.org/content/10.1101/2020.02.12.941716v1.full. 10.1101/2020.02.12.941716. 10.1186/s12915-020-00862-9 PMC7570057 33070780

[77] Wu C, Jordan MD, Newcomb RD, Gemmell NJ, Bank S, Meusemann K (2017). Analysis of the genome of the New Zealand giant collembolan (Holacanthella duospinosa) sheds light on hexapod evolution. BMC Genomics.

[78] Brand P, Lin W, Johnson BR (2018). The draft genome of the invasive walking stick, Medauroidea extradendata, reveals extensive lineage-specific gene family expansions of cell wall degrading enzymes in Phasmatodea. G3 genes, genomes. Genet.

[79] Faddeeva-Vakhrusheva A, Derks MFL, Anvar SY, Agamennone V, Suring W, Smit S (2016). Gene family evolution reflects adaptation to soil environmental stressors in the genome of the collembolan Orchesella cincta. Genome Biol Evol.

[80] Johnston JS, Yoon KS, Strycharz JP, Pittendrigh BR, Clark JM (2007). Body lice and head lice (Anoplura: Pediculidae) have the smallest genomes of any hemimetabolous insect reported to date. J Med Entomol.

[81] Grishin NV, Shen J, Cong Q, Kinch LN, Borek D, Otwinowski Z (2016). Complete genome of *Pieris rapae*, a resilient alien, a cabbage pest, and a source of anti-cancer proteins. F1000Research.

[82] Kim HS, Murphy T, Xia J, Caragea D, Park Y, Beeman RW (2009). BeetleBase in 2010: revisions to provide comprehensive genomic information for Tribolium castaneum. Nucleic Acids Res.

[83] Battelle B-A, Ryan JF, Kempler KE, Saraf SR, Marten CE, Warren WC (2016). Opsin repertoire and expression patterns in horseshoe crabs: evidence from the genome of Limulus polyphemus (Arthropoda: Chelicerata). Genome Biol Evol.

