# Supplementary material for: Multi-faceted analysis provides little evidence for recurrent whole-genome duplications during hexapod evolution
Source: BMC Biol. 2020 May 27;18:57. doi: 10.1186/s12915-020-00789-1 (PMC7251882; doi:10.1186/s12915-020-00789-1)
Supplement: Supplementary file 1 — Additional file 1: Figure S1. Ks frequency distribution graphs and circos plots of collinear syntenic blocks for gene duplicates in the genomes of A Aedis aegypti, B Acyrthosiphon pisum, C Apis mellifera, D Athalia rosae, E Bemisia tabaci, F Blattella germanica, G Campodea augens, H Drosophila melanogaster, I Frankliniella occidentalis, J Holacanthella duospinosa, K Medauroidea extradentata, L Orchesella cincta, M Pediculus humanus, N Pieris rapae, O Tribolium castaneum. Orange-red, frequency distribution of gene duplicate bins with identical Ks values; light blue, WGD/segmental duplication event predicted by MSscanX; inlay, circos plot co-linear blocks. Figure S2. Histograms of sequence read coverage distribution (bins of 20 counts) among scaffolds of Aethina tumida’s genome assembly: 1000 random contigs (A) and contigs with co-linear regions (B). Figure S3. Scatter plots of putative gene duplicates (BlastP hits with e-value < 10–10) for species that contain at least one segmental duplication. A) L. polyphemus, b) A. aegypti c) A. tumida, d) B. germanica, e) B. mori, f) C. felis, g) F. candida. Co-linear blocks identified by MCScanX are indicated as red dots. Scale on horizontal axis in bp. Figure S4. Trace plots for the MCMC samples for the Holometabola data set with the IR prior. In black results for the full data set are shown (10,000 generations after 1000 generations as burn-in, showing every iterate), whereas the other transparent colors show three replicate chains for a random subset of 1000 gene families (20,000 generations after 1000 as burn-in, showing every second iterate). Duplication (λ) and loss (μ) rates are shown on a log10 scale, and subscripts denote branches of the species tree. Figure S5. Marginal posterior distributions for the MCMC samples for the Holometabola data set with the IR prior. Interpretation is as in Figure S4, but here we show the rates on the original scale. Figure S6. Marginal posterior distributions for retention rates (q) of th [file 12915_2020_789_MOESM1_ESM.pdf]

Supplemental information

Figure S1 Ks frequency distribution graphs and circos plots of collinear syntenic blocks for gene duplicates in the genomes of **A** *Aedis aegypti,* **B** *Acyrthosiphon pisum*, **C** *Apis mellifera*, **D** *Athalia rosae*, E *Bemisia tabaci*, F *Blattella germanica*, G *Campodea augens*, H *Drosophila melanogaster*, I *Frankliniella occidentalis*, J *Holacanthella duospinosa*, K *Medauroidea extradentata*, L *Orchesella cincta*, M *Pediculus humanus*, N *Pieris rapae*, O *Tribolium castaneum*. Orange-red, frequency distribution of gene duplicate bins with identical Ks values; light-blue, WGD/segmental duplication event predicted by MSscanX; inlay, circos plot co-linear blocks.

Figure S2 Histograms of sequence read coverage distribution (bins of 20 counts) among scaffolds of *Aethina tumida's* genome assembly: 1000 random contigs (A) and contigs with co-linear regions (B).

Figure S3. Scatter plots of putative gene duplicates (BlastP hits with e-value < 10^{-10}) for species that contain at least one segmental duplication. A) *L. Polyphemus*, b) *A. aegypti* c) *A. tumida*, d) *B. germanica*, e) *B. mori*, f) *C. felis*, g) *F. candida*. Co-linear blocks identified by MCScanX are indicated as red dots. Scale on horizontal axis in bp.

Figure S4 Trace plots for the MCMC samples for the Holometabola data set with the IR prior. In black results for the full data set are shown (10000 generations after 1000 generations as burn-in, showing every iterate), whereas the other transparent colors show three replicate chains for a random subset of 1000 gene families (20000 generations after 1000 as burn-in, showing every second iterate). Duplication (λ) and loss (μ) rates are shown on a $\log_{10}$ scale, and subscripts denote branches of the species tree.

Figure S5 Marginal posterior distributions for the MCMC samples for the Holometabola data set with the IR prior. Interpretation is as in Figure S4, but here we show the rates on the original scale.

Figure S6 Marginal posterior distributions for retention rates (q) of the five hypothetical WGD events marked along the Holometabola tree. The upper row shows results under the IR prior, whereas the lower row corresponds to results under the GBM (autocorrelated rates) prior (see methods). Note that the distributions under the GBM prior for the Lepidoptera, Coleoptera and Hymenoptera events are vanishingly small but are shown on the same scale as the upper row for the sake of comparison.

Figure S7 A distinct mode for the parameters associated with the *C. felis* branch was observed in one of the chains under the IR prior for the Holometabola tree, indicating the possible problem of inefficient sampling of multimodal distributions in Whale. Results from three independent chains are shown in blue, orange and green respectively. (a & d) Marginal posterior distributions for the duplication (λ) and retention (q) rate associated with the *C. felis* branch for two chains. (b,c & e) Trace plots for duplication,

loss (μ) and retention rates associated with the *C. felis* branch for the same two chains. (f) Trace plot of the log likelihood for these chains.

Figure S8 Posterior reconciliation probabilities of gene duplicates reconciled to the hypothetical *C. felis* (A) or Insecta (B) WGDs. The posterior reconciliation probability is calculated as the fraction that a particular clade is reconciled to the WGD node of interest in 1000 reconciled trees sampled from the posterior. Boxplots show the same data but grouped by clade size, showing for the *C. felis* WGD hypothesis a slight trend towards lower reconciliation probabilities for larger clades, whereas this trend is not observed for the putative Insecta event.

Figure S9 Trace plots for the MCMC samples for the non-Holometabola data set with the IR prior. In black results for the full data set are shown (10000 generations after 1000 generations as burn-in, showing every iterate), whereas the other transparent colors show three replicate chains for a random subset of 1000 gene families (20000 generations after 1000 as burn-in, showing every second iterate). Duplication (λ) and loss (μ) rates are shown on a $\log_{10}$ scale and subscripts denote branches of the species tree.

Figure S10 Marginal posterior distributions for the MCMC samples for the non-Holometabola data set with the IR prior. Interpretation is as in Figure S8 but here we show the rates on the original scale.

Figure S11 Marginal posterior distributions for retention rates (q) of the seven hypothetical WGD events marked along the non-Holometabola tree. The upper row shows results under the IR prior, whereas the lower row corresponds to results under the GBM (autocorrelated rates) prior (see methods). Note that the distributions under the GBM prior for the Colembolla and Polyneoptera events are vanishingly small but are shown on the same scale as the upper row for the sake of comparison.

Table S1 General specifications of species included in this study. Gene pairs, the number of gene pairs per hexapod species used as input for Ks calculation, co-linearity analysis and gene tree-species tree reconciliation analysis. Gene pairs with Ks values of 0 and higher than 5 were filtered out.

Figure S1

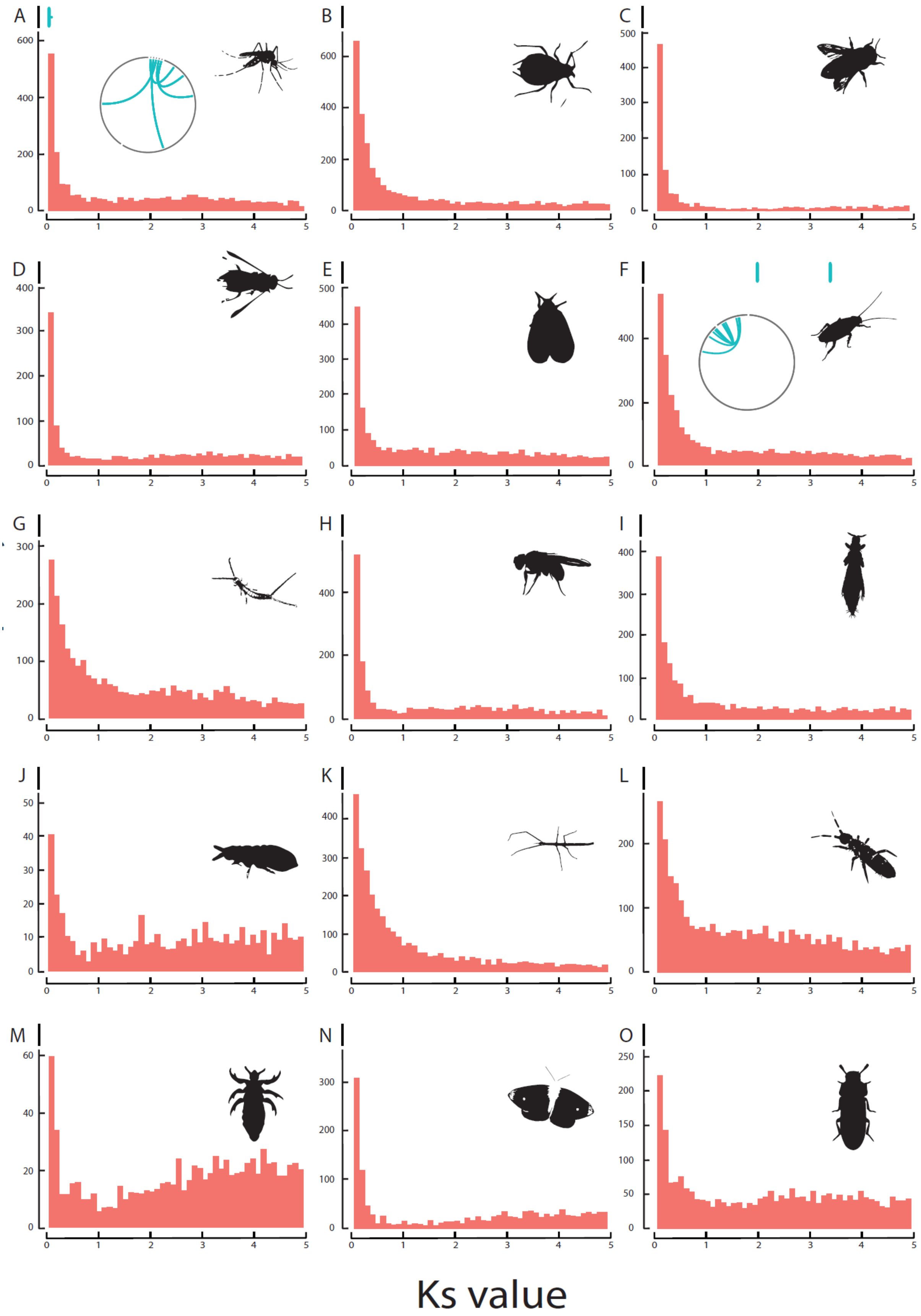

Figure S2

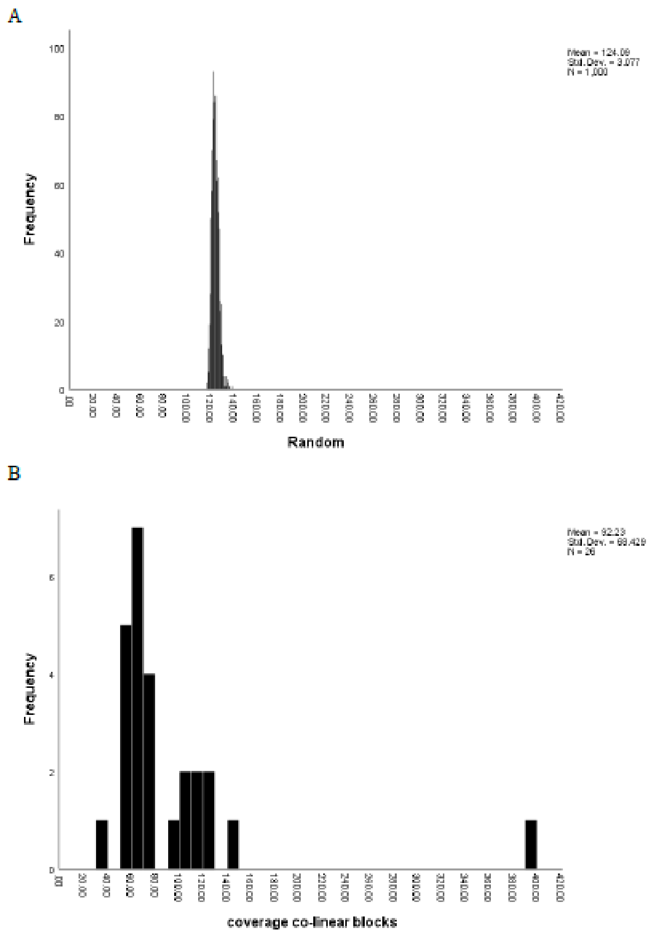
A
Mean = 124.09
Std.Dev. = 3.077
N = 1,000
Frequency
Random
B
Mean = 92.23
Std.Dev. = 68.429
N = 26
Frequency
coverage co-linear blocks

Figure S3

A)

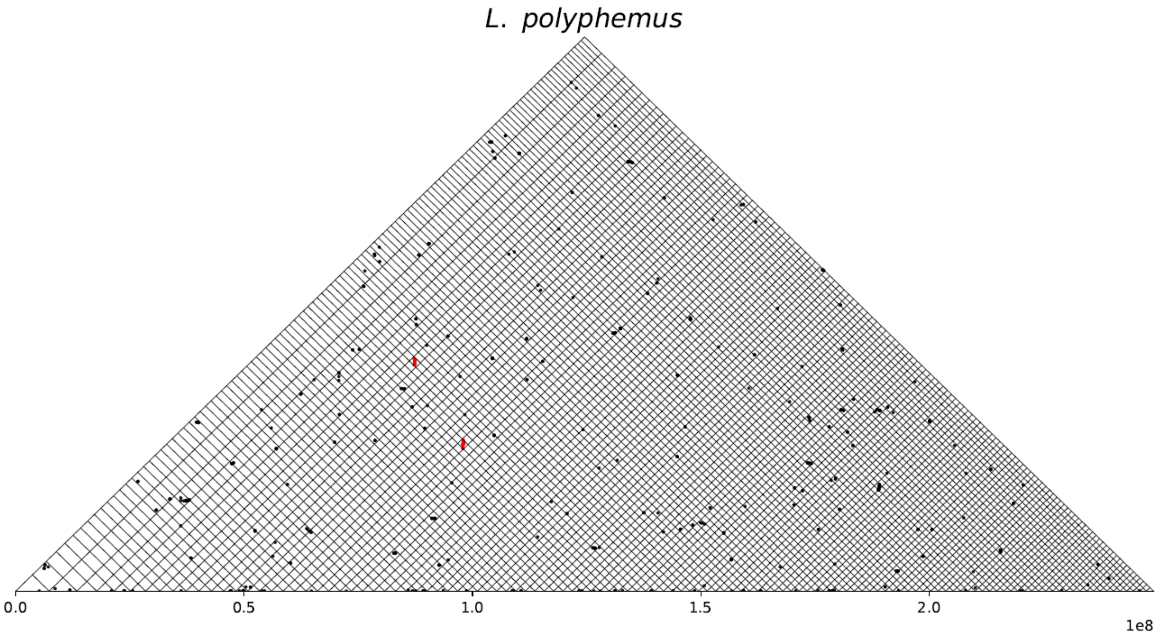
L. polyphemus
0.0
0.5
1.0
1.5
2.0
1e8

B)

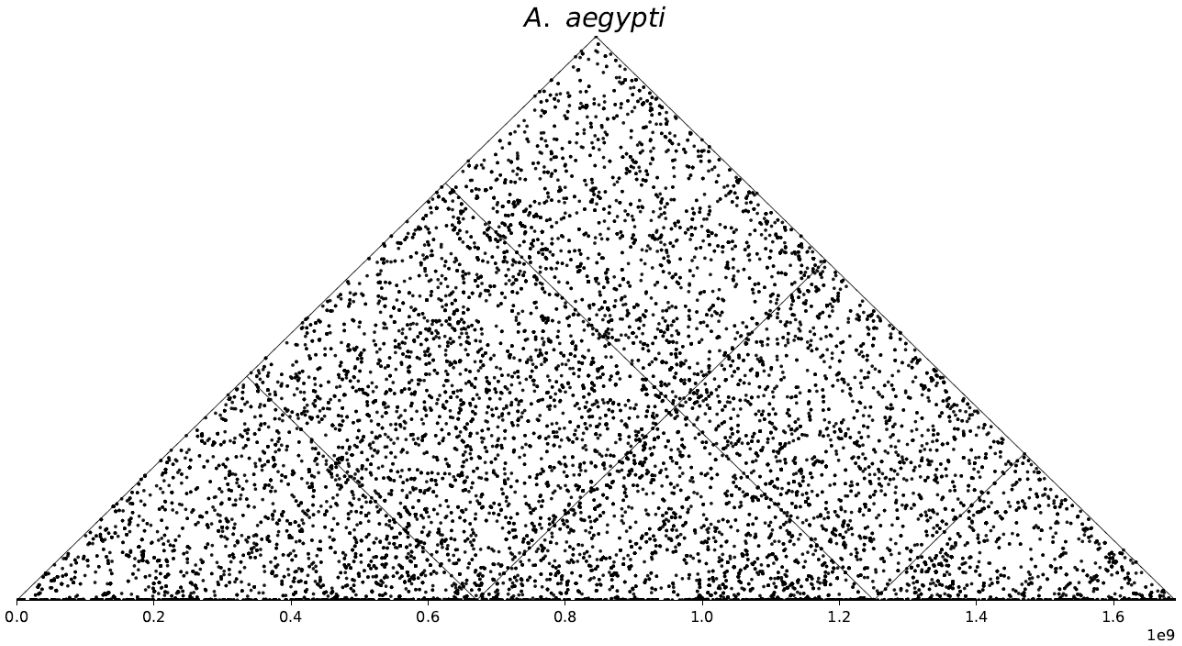
A. aegypti
0.0
0.2
0.4
0.6
0.8
1.0
1.2
1.4
1.6
1e9

C)

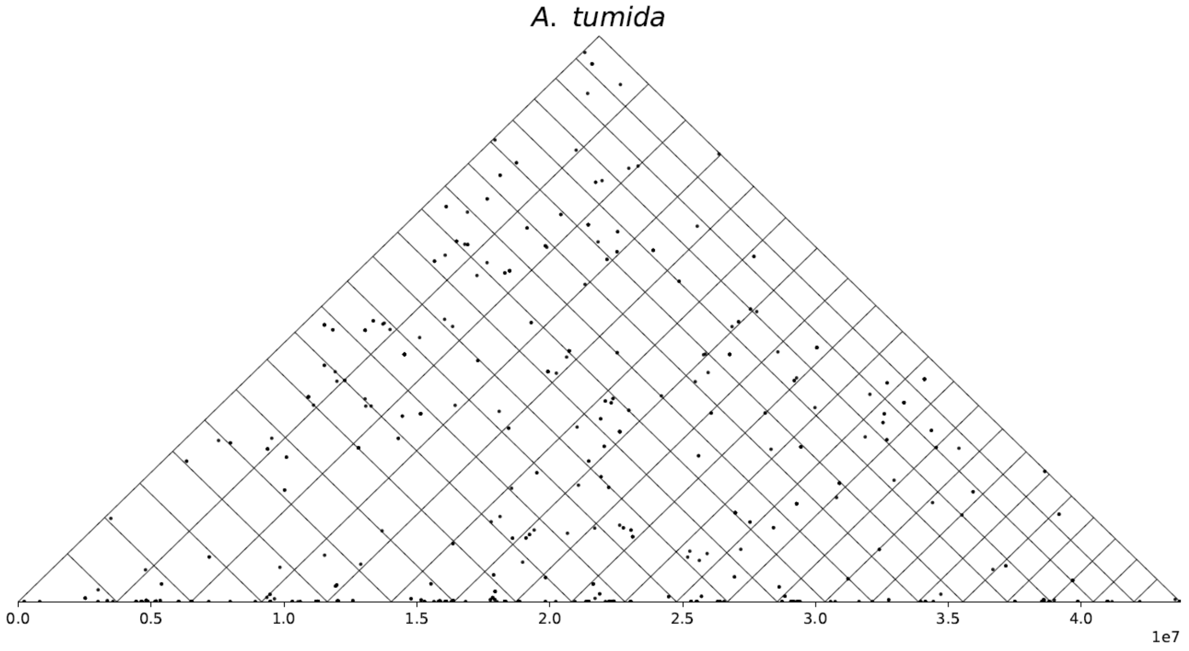
A. tumida
0.0
0.5
1.0
1.5
2.0
2.5
3.0
3.5
4.0
1e7

D)

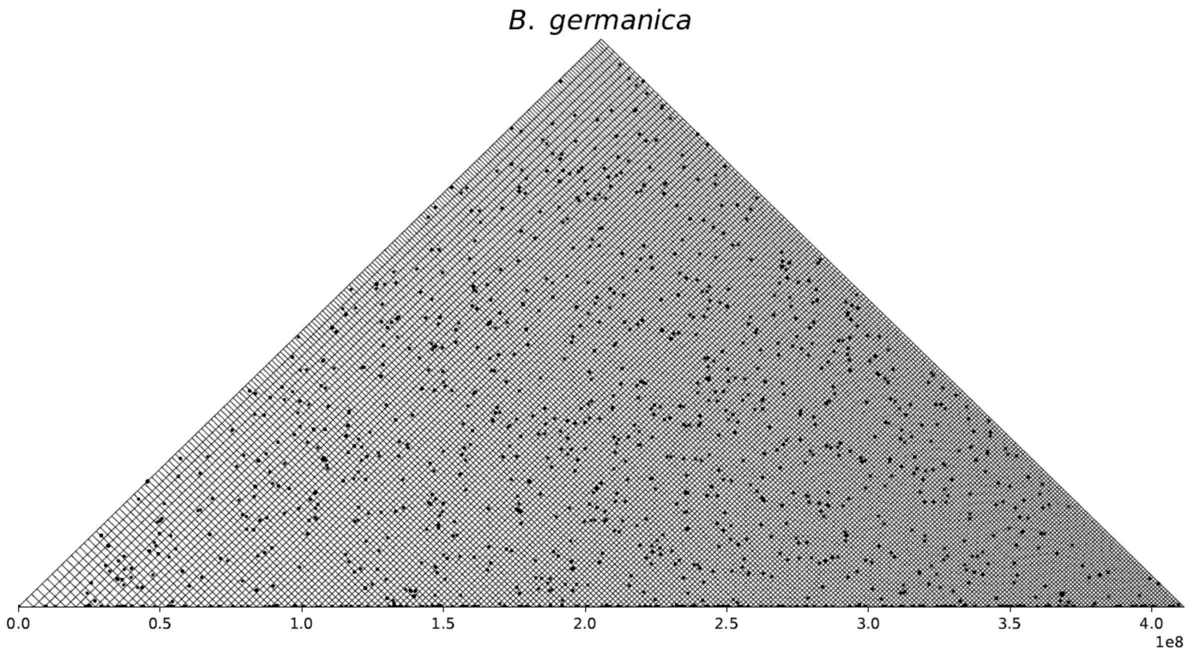
B. germanica
0.0
0.5
1.0
1.5
2.0
2.5
3.0
3.5
4.0
1e8

E)

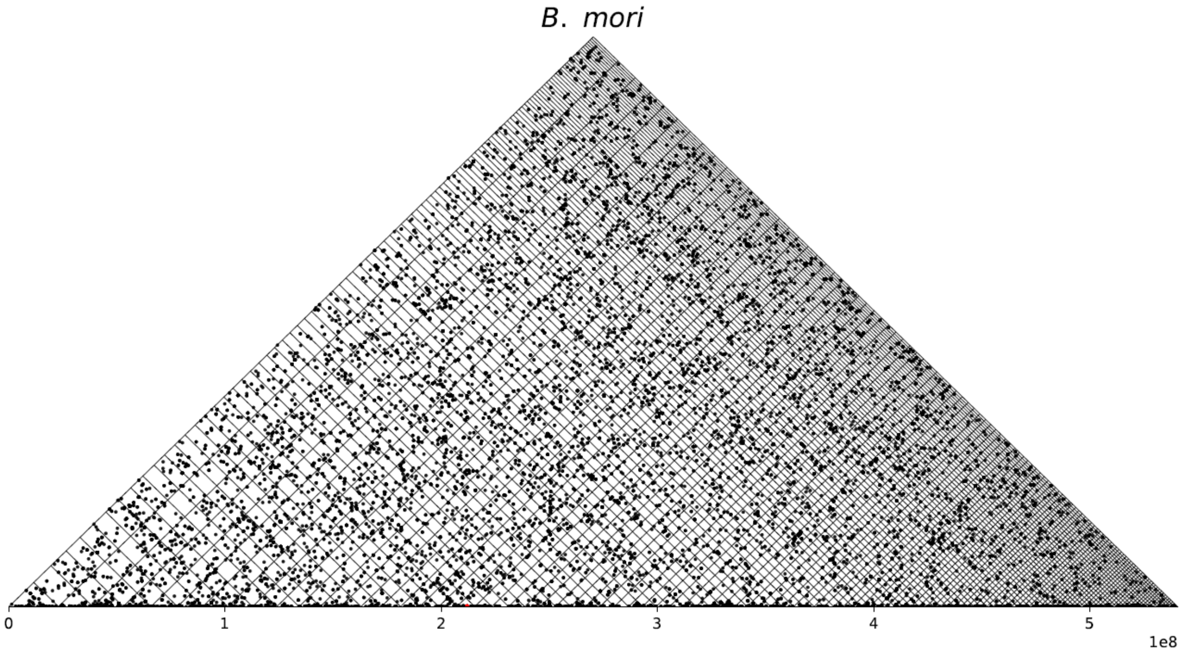

F)

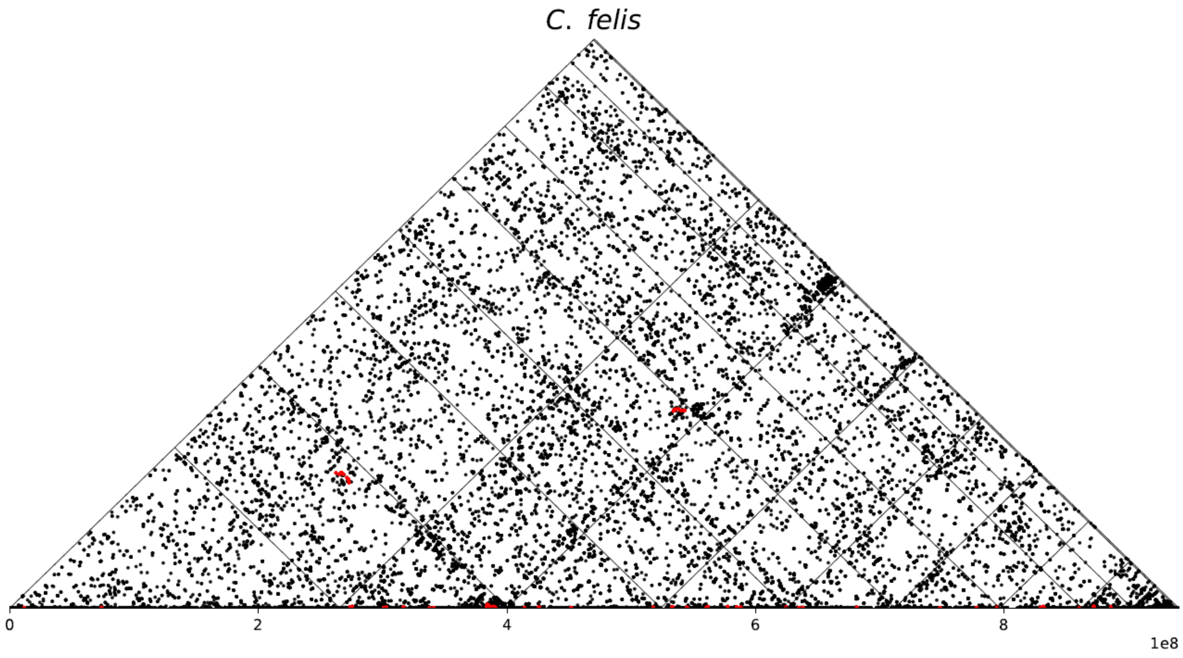

G)

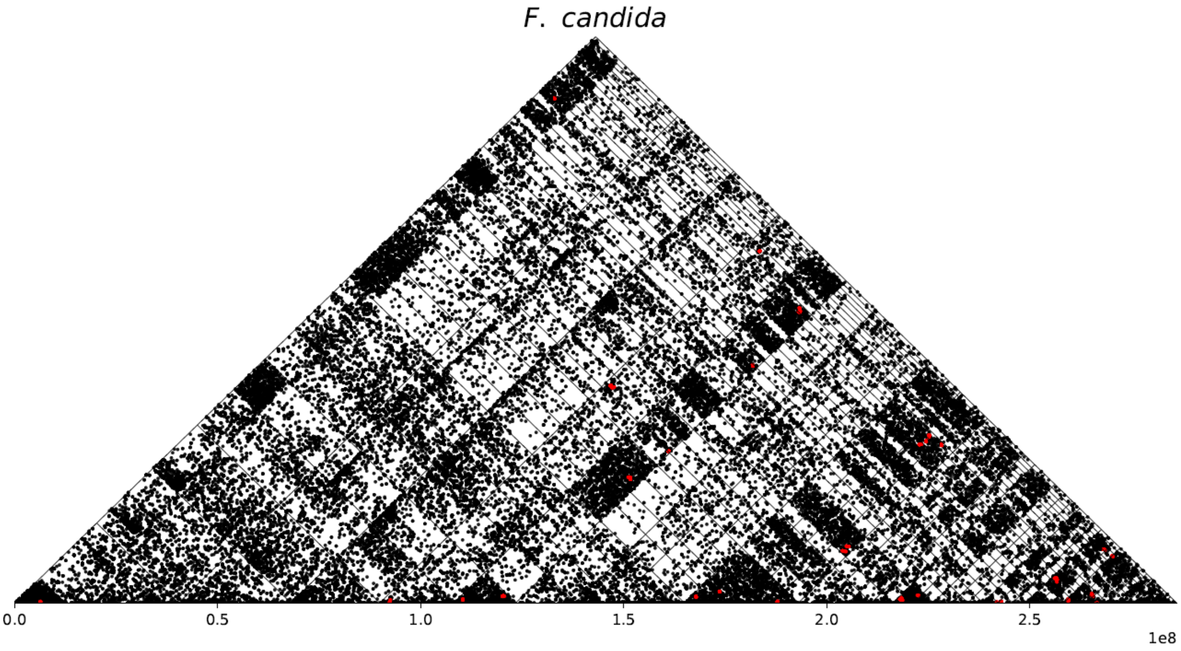

Figure S4

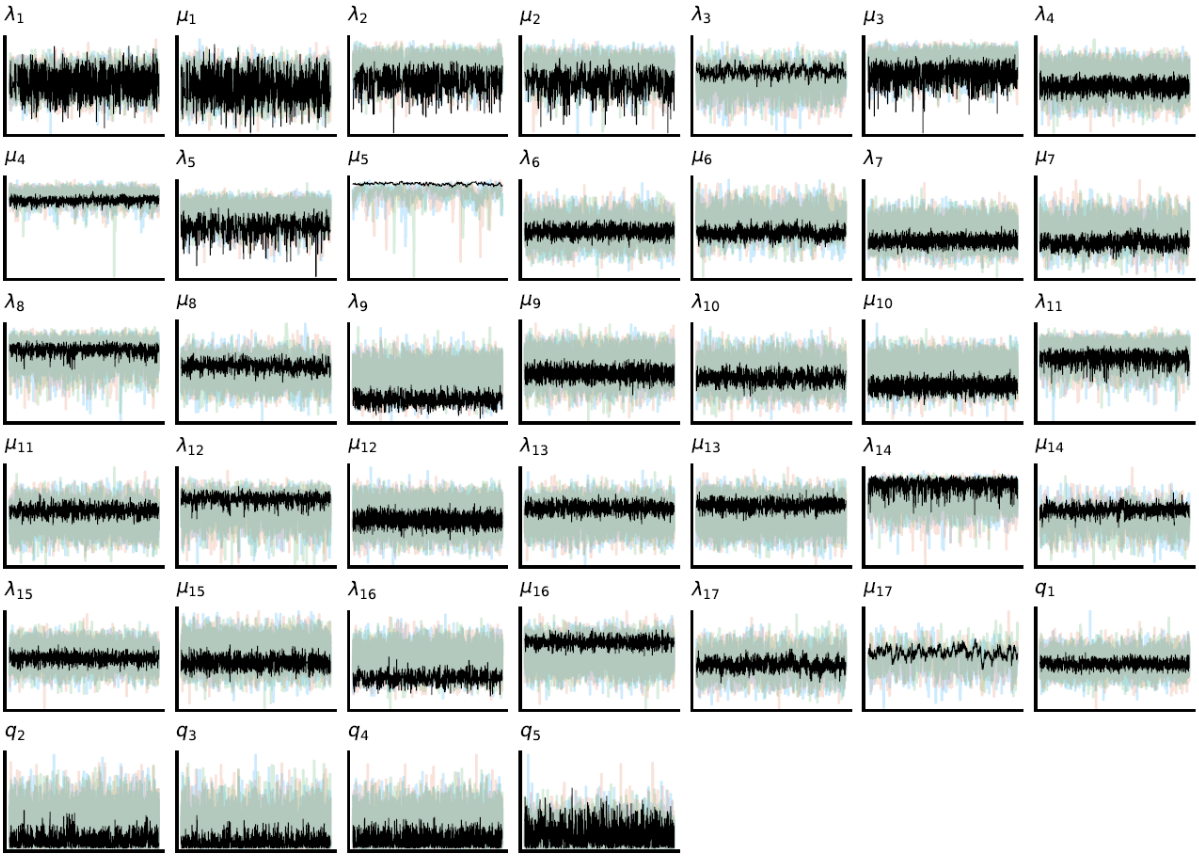

λ_1
μ_1
λ_2
μ_2
λ_3
μ_3
λ_4
μ_4
λ_5
μ_5
λ_6
μ_6
λ_7
μ_7
λ_8
μ_8
λ_9
μ_9
λ_{10}
μ_{10}
λ_{11}
μ_{11}
λ_{12}
μ_{12}
λ_{13}
μ_{13}
λ_{14}
μ_{14}
λ_{15}
μ_{15}
λ_{16}
μ_{16}
λ_{17}
μ_{17}
q_1
q_2
q_3
q_4
q_5

Figure S5

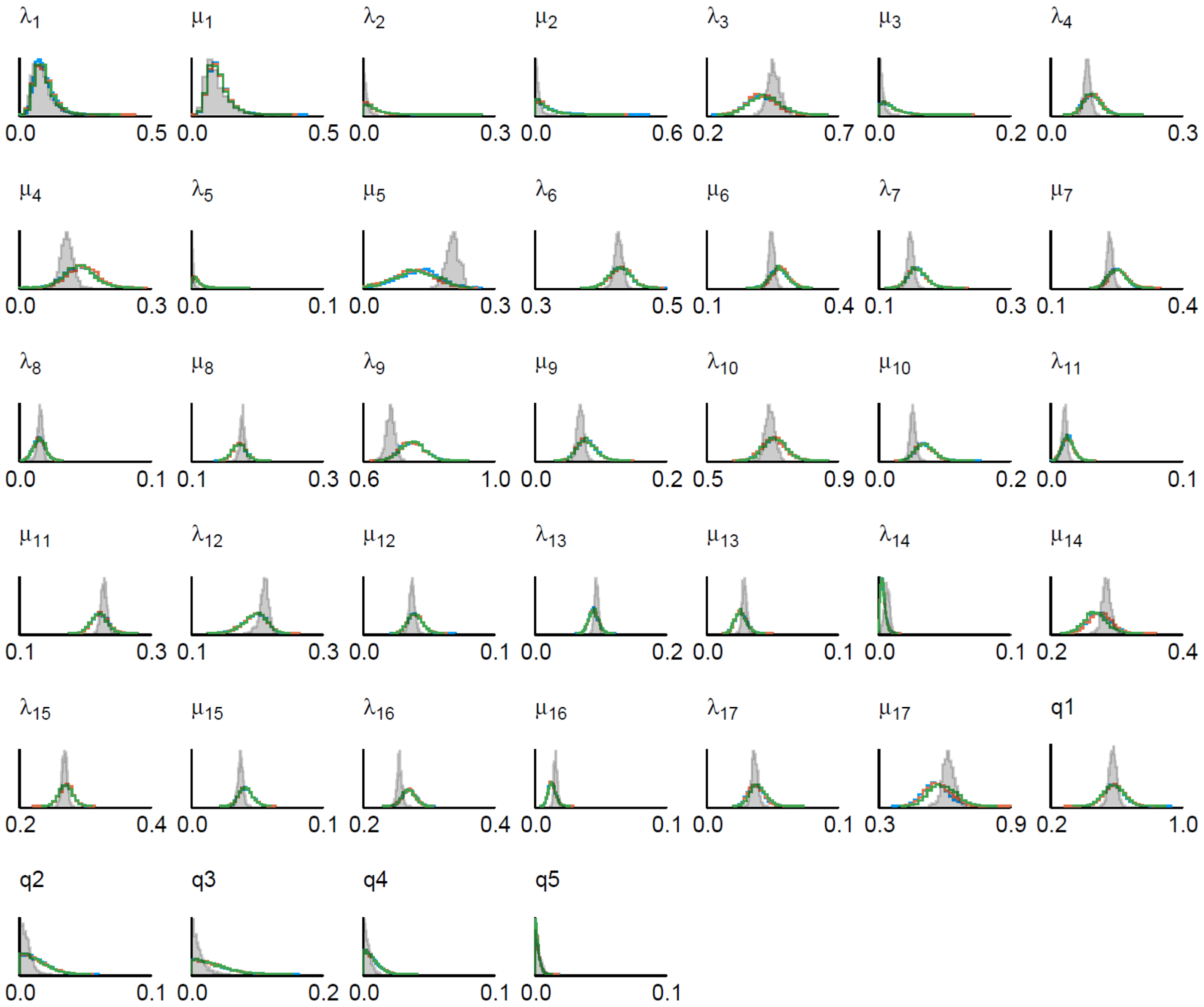
λ1
0.0 0.5
μ1
0.0 0.5
λ2
0.0 0.3
μ2
0.0 0.6
λ3
0.2 0.7
μ3
0.0 0.2
λ4
0.0 0.3
μ4
0.0 0.3
λ5
0.0 0.1
μ5
0.0 0.3
λ6
0.3 0.5
μ6
0.1 0.4
λ7
0.1 0.3
μ7
0.1 0.4
λ8
0.0 0.1
μ8
0.1 0.3
λ9
0.6 1.0
μ9
0.0 0.2
λ10
0.5 0.9
μ10
0.0 0.2
λ11
0.0 0.1
μ11
0.1 0.3
λ12
0.1 0.3
μ12
0.0 0.1
λ13
0.0 0.2
μ13
0.0 0.1
λ14
0.0 0.1
μ14
0.2 0.4
λ15
0.2 0.4
μ15
0.0 0.1
λ16
0.2 0.4
μ16
0.0 0.1
λ17
0.0 0.1
μ17
0.3 0.9
q1
0.2 1.0
q2
0.0 0.1
q3
0.0 0.2
q4
0.0 0.1
q5
0.0 0.1

Figure S6

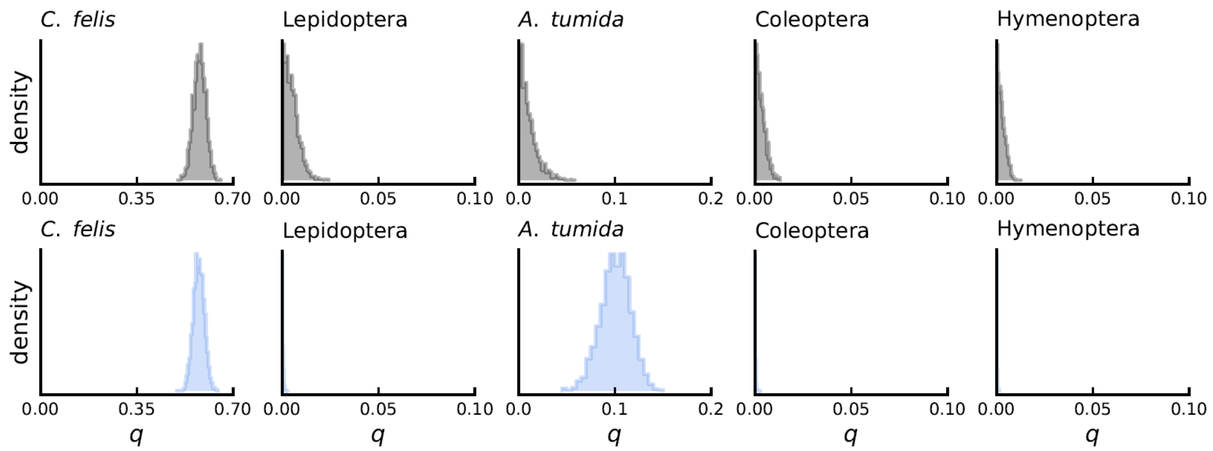

Figure S7

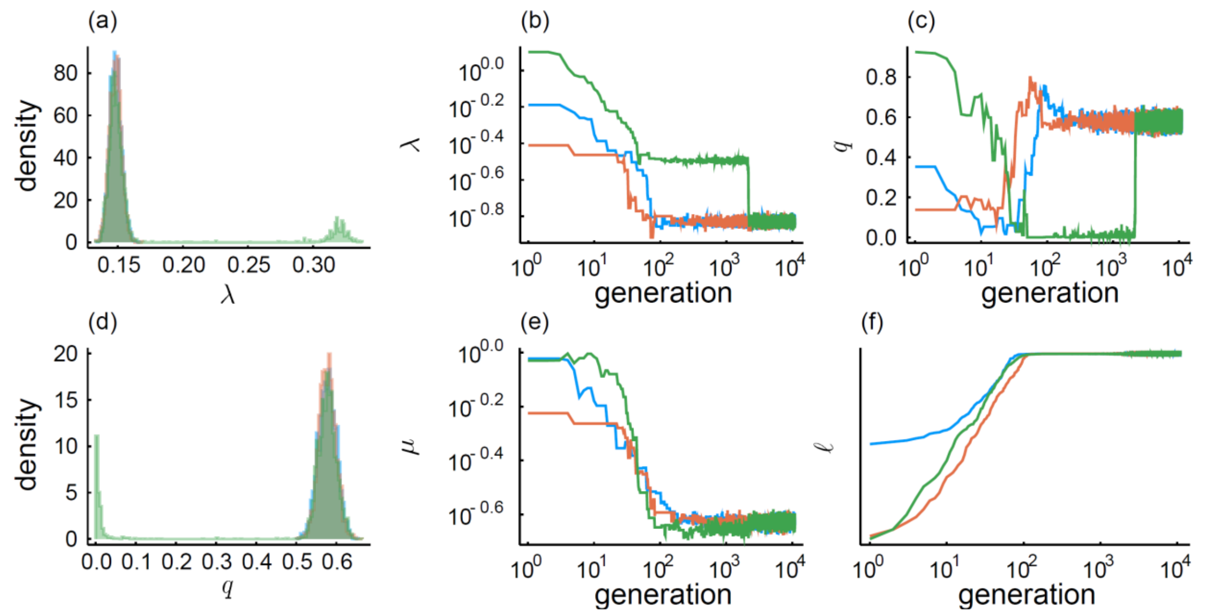

Figure S8

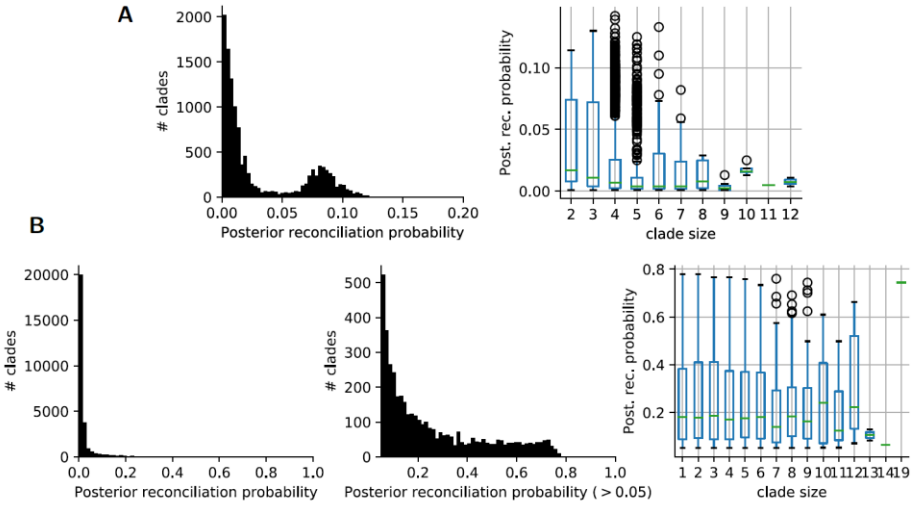

Figure S9

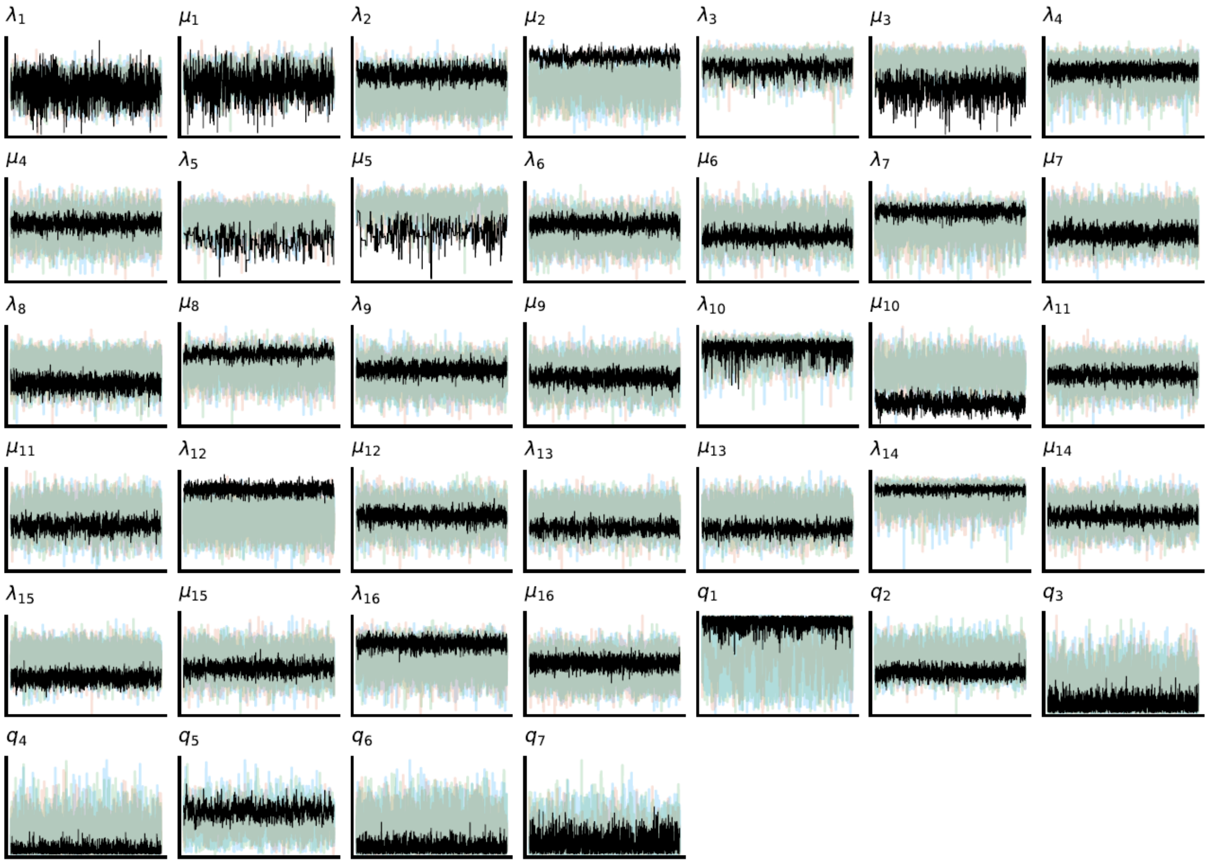

Figure S10

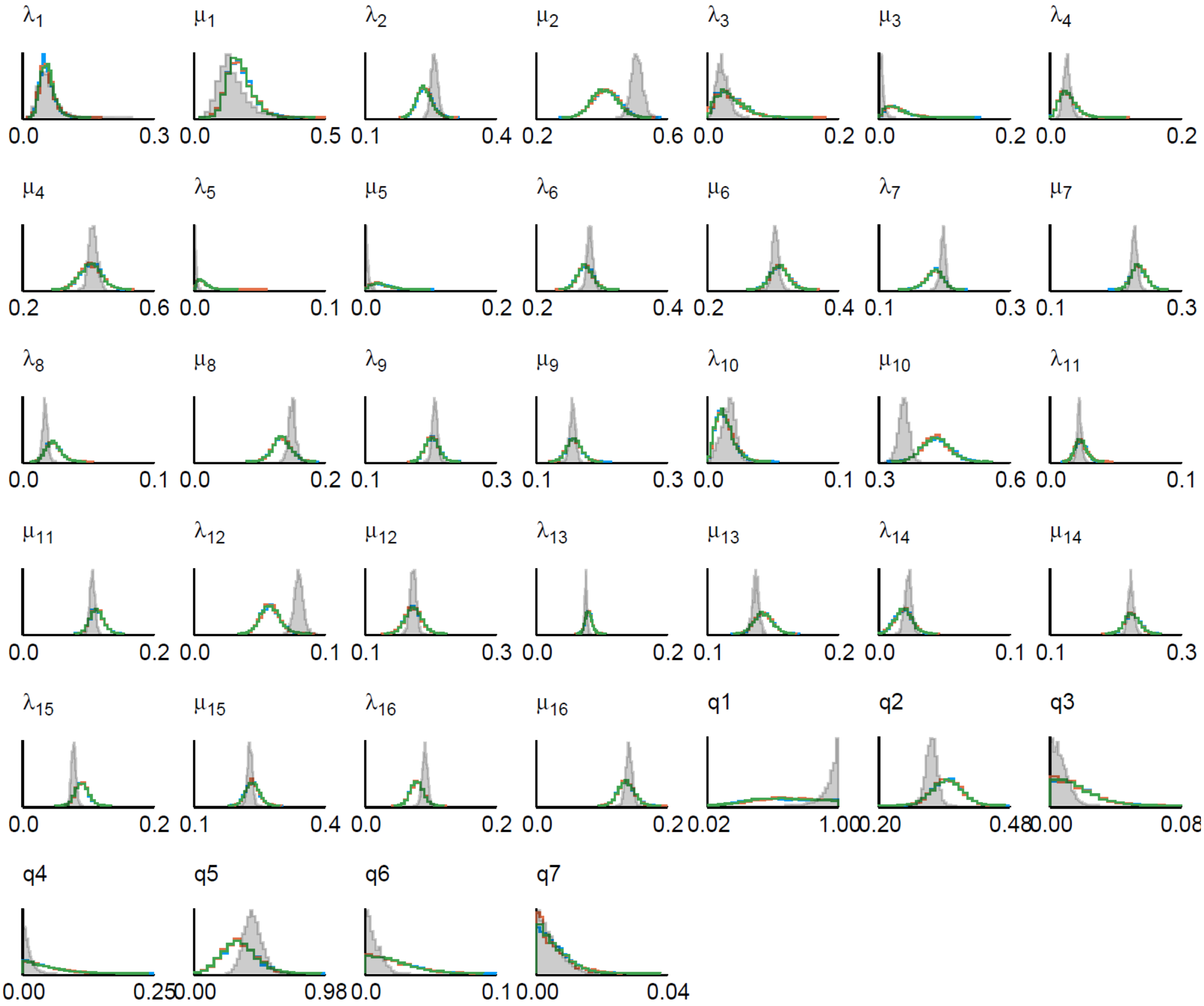

λ1 0.0 0.3
μ1 0.0 0.5
λ2 0.1 0.4
μ2 0.2 0.6
λ3 0.0 0.2
μ3 0.0 0.2
λ4 0.0 0.2
μ4 0.2 0.6
λ5 0.0 0.1
μ5 0.0 0.2
λ6 0.2 0.4
μ6 0.2 0.4
λ7 0.1 0.3
μ7 0.1 0.3
λ8 0.0 0.1
μ8 0.0 0.2
λ9 0.1 0.3
μ9 0.1 0.3
λ10 0.0 0.1
μ10 0.3 0.6
λ11 0.0 0.1
μ11 0.0 0.2
λ12 0.0 0.1
μ12 0.1 0.3
λ13 0.0 0.2
μ13 0.1 0.2
λ14 0.0 0.1
μ14 0.1 0.3
λ15 0.0 0.2
μ15 0.1 0.4
λ16 0.0 0.2
μ16 0.0 0.2
q1 0.02 1.00
q2 0.20 0.48
q3 0.00 0.08
q4 0.00 0.25
q5 0.00 0.98
q6 0.0 0.1
q7 0.00 0.04

Figure S11

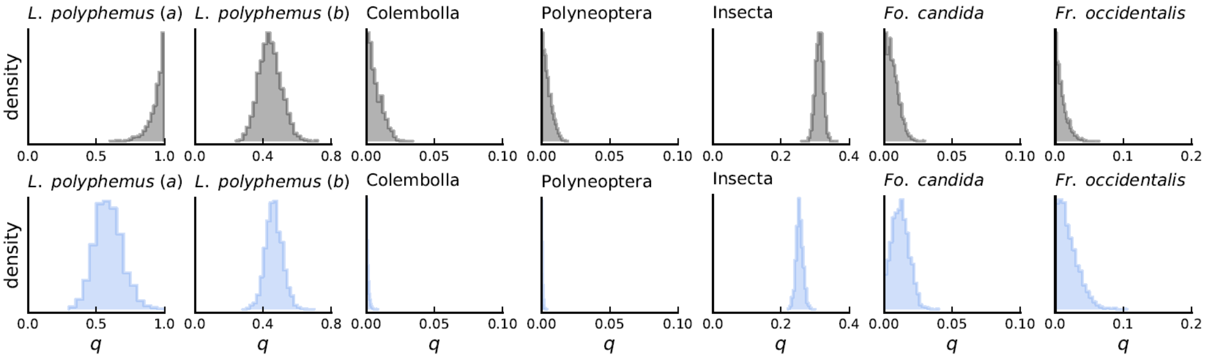
L. polyphemus (a)
L. polyphemus (b)
Colembolla
Polyneoptera
Insecta
Fo. candida
Fr. occidentalis
density
q

Table S1.

Species	Gene pairs	Segmental duplications	Genome size (Mb)	Protein count	No. of scaffolds	Sequencing technology	Accession number with literature reference in square brackets
Acyrthosiphon pisum	14446	0	541	27999	23925	Sanger	GCF_000142985.2 [48]
Aedes aegypti	21073	5	1278	28317	2310	Sanger/10X, Hi-C	GCF_002204515.2 [68]
Aethina tumida	9700	13	234	17463	3063	Illumina HiSeq; PacBio	GCF_001937115.1 [69]
Apis mellifera	9666	0	235	22456	5321	Sanger/ABI Solid/Roche 454	GCF_000002195.4 [70]
Athalia rosae	8967	0	156	22140	522	Illumina mate-pair/pair end	GCF_000344095.1 [71]
Bemisia tabaci	14057	0	636	22737	19751	Illumina mate-pair/pair-end	GCF_001854935.1 [72]
Blattella germanica	19308	1	1916	26325	28065	Illumina mate-pair/pair-end	GCA_000762945.2 [46]
Bombyx mori	8936	2	397	19618	43463	Sanger	GCF_000151625.1 [73]
Campodea augens	7710	0	1130	23978	18761	Illumina mate-pair/pair-end	campodea_augens_genome_v1.0 [67]
Ctenocephalides felis	12961	49	775	21954	3733	PacBio Sequel	GCF_003426905.1 [74]
Drosophila melanogaster	15975	0	138	30482	1870	Sanger/PacBio SMRT	GCF_000001215.4 [75]
Folsomia candida	20663	55	222	28734	162	PacBio SMRT	fcand_genome.fa (Collembolomics.nl) [35]
Frankliniella occidentalis	11584	0	275	23356	18479	Illumina mate-pair/pair-end	GCF_000697945.2 [76]
Holacanthella duospinosa	1225	0	327	9895	62430	Illumina mate-pair/pair-end	GCA_002738285.1 [77]
Medauroidea extradentata	11715	0	2593	35797	135691	Illumina mate-pair/pair-end	GCA_003012365.1 [78]
Orchesella cincta	8726	0	287	20249	9402	Illumina HiSeq; PacBio SMRT	ocinc_genome.fa (Collembolomics.nl) [79]
Pediculus humanus	3462	0	111	10775	1882	Sanger	GCF_000006295.1 [80]
Pieris rapae	7986	0	246	18979	7349	Illumina mate-pair/pair-end	GCF_001856805.1 [81]
Tribolium castaneum	8927	0	166	18536	2081	Sanger/Illumina mate-pair	GCA_000002335.3 [82]
Zootermopsis nevadensis	4914	0	485	14610	31663	Illumina pair-end	GCA_000696155.1 [47]
Limulus polyphemus	31186	7	1828	38682	286793	Roche 454	GCF_000517525.1 [83]

Footnote. Number of gene pairs and number of segmental duplications was not significantly correlated with assembly errors (Pearson correlation, P=0.08). Also, PacBio generated genomes yielded both high numbers of segmental duplications (e.g. *F. candida*) as well as no segmental duplications (e.g. *O. cincta*).
